# Investigating the effects of ICT, education, and R&D on economic efficiency and technology heterogeneity: A cross-country analysis

**DOI:** 10.1016/j.heliyon.2024.e28168

**Published:** 2024-03-19

**Authors:** Rashid S. Aljneibi, Panagiotis D. Zervopoulos, Angelos Kanas

**Affiliations:** a University of Sharjah, College of Business Administration, Sharjah, United Arab Emirates; b University of Sharjah, Department of Management, Sharjah, United Arab Emirates; c University of Piraeus, Department of Economics, Piraeus, Greece

**Keywords:** Education, ICT, R&D, Efficiency, Bayesian methods

## Abstract

This study explores the impact of information and communication technology (ICT), education, and research and development (R&D) on countries' economic efficiency and technology heterogeneity. A panel of 52 countries, classified into developed (31 sample countries) and developing (21 sample countries) during 2011–2019, were the data sources for the analysis. We obtained relative country-level economic efficiency and technology gap inefficiency estimates from a novel Bayesian data envelopment analysis (DEA) approach. Bias-corrected estimates yielded by this technique have proven to be valid. We then regressed the estimates on ICT, education, and R&D proxy variables using a two-step and an iterative generalized method of moments (GMM) for linear dynamic panel data. Our analysis mitigates possible feedback effects between the explanatory and response variables, as well as possible endogeneity. The novelty of this work goes beyond the use of a new data analysis technique, investigating the impact of the three factors mentioned above and the country classification on technology heterogeneity caused by differences in countries' market structures, regulatory frameworks, economic and knowledge ecosystems, and cultures. Based on this study's findings, fixed broadband subscriptions have the most significant effect on economic efficiency improvement, while R&D is the main driver for reducing technology gap inefficiency. Specifically, ICT-facilitated knowledge spillovers within and across country groups through R&D cooperation play a significant positive role in closing the technology gap. This finding is consistent with the catch-up theory.

## 1 Introduction

Information and communication technology (ICT), such as the Internet and mobile phone technologies, has transformed how individuals, firms, and countries work, communicate, and behave. [[1], [2], [3], [4]], and [5] highlighted the crucial role that ICT plays in productivity improvement and economic growth [[6], [7], [8]]. argued that ICT reduces productivity gaps between developed and developing countries. However, the magnitude of ICT impact on economic growth and productivity varies depending on a country's economic development level ([7,9,10]), the penetration of ICT into economic activities ([[11], [12], [13]]), the development of a country's human capital ([[14], [15], [16]]) and the investments in research and development (R&D) ([17,18]).

[14,15] studied the positive effects of education on economic growth and productivity. Also [19], revealed the link between education and innovation, while [17] discussed the critical role of R&D in innovations. Education is critical for human capital development ([16]). It is clear overall that ICT, education, and R&D can predict economic growth. For instance Ref. [20], pointed out that educated people can make better use of ICT, such as the Internet and mobile services, and a skilled workforce reinforces R&D [21,22]. identified the complementarity between employees’ educational levels and R&D. It is noteworthy that neoclassical economists have argued that the relationship between knowledge and economic growth is linear ([14,15]).

On the other hand, evolutionary economists support a conditional relationship between the two factors depending on exogenous social and institutional variables ([23]) [24]. studied the joint effect of knowledge, emphasizing the roles of tertiary educated people and R&D on economic growth, establishing the significant positive contribution of both factors. According to the same study, although a population with a tertiary education benefits economic activity regardless of a region's development, R&D investments benefit more developed regions than developing ones. However [6], argued that developed and developing countries' economic prosperity converges over time because of ICT.

The discussion of the conditional impact of ICT, education, and R&D on economic activity and the convergence of economies of developing countries with those of more developed countries is central to the catch-up theory ([23,25]). According to this theory, developing countries leverage the experiences of developed nations to enhance their economic efficiency and close the gap with the latter countries, embarking on a learning curve.

The main body of the literature has discussed the impact of some of the three dimensions we incorporate in our analysis on economic growth or productivity (e.g., education and R&D, education and ICT, ICT and R&D), but not all three at once, along with country classification. Also, to the best of our knowledge, this is the first study to elaborate on the effects of ICT, education, and R&D on countries' technology heterogeneity. Technology heterogeneity, or technology gap inefficiency, typically depends on factors such as national market structure characteristics, national regulatory framework, economic and knowledge ecosystems, and culture. We use a novel Bayesian data envelopment analysis (DEA) approach to estimate country-specific efficiencies and technology gap inefficiencies. This technique yields valid estimates that can improve the quality of this study's outcomes compared with those of studies using conventional efficiencies, which are more likely to be biased ([[26], [27], [28]]). The efficiency and technology gap inefficiency estimates are regressed on the three dimensions mentioned above using a two-step and iterative GMM for linear dynamic panel data, which yield average fixed-effect coefficients for the regression models' explanatory variables. This study's regression models use Bayesian DEA estimates to mitigate possible feedback effects between the explanatory and response variables. Drawing on the above discussion, this work's novel contributions are both methodological and empirical, providing information to policymakers about country-level economic efficiency improvement and technology heterogeneity mitigation through ICT, education, and R&D.

The remainder of this study is organized as follows. Section 2 discusses the literature on the impacts of ICT, education, and R&D investments on economic activity. Section 3 provides details of the methodology—i.e., the Bayesian DEA approach, the regression models, and the dataset. Section 4 discusses the empirical analysis results. Section 5 concludes the paper.

## 2 Literature review

The effect of ICT on economic growth and productivity has been thoroughly studied in the literature ([29]). Specifically [18,30], stated that ICT contributed positively to productivity and economic growth in the GDP of developing nations. According to Ref. [31], the most important sources of labor productivity growth in the industrial sectors in most developing countries, such as India, are investments in ICT and foreign R&D spillovers in the ICT sector. In their research [32], investigated the impact of ICT investment on worker productivity and GDP in Poland. They discovered that ICT investment contributed 8.9% to GDP and 12.7% to labor productivity growth. In line with the above studies [30], indicated that the growing use of ICT aided the restructuring of the manufacturing sector in Central and Eastern Europe and the subsequent convergence process of these nations with the former EU-15.

Moreover [33], investigated the influence of ICT investment on labor productivity and total factor productivity in Japan. They discovered that the Japanese economy had seen significant economic growth in total factor productivity (TFP) compared to the EU's four largest economies (Germany, France, the United Kingdom, and Italy) because of ICT investment. According to Ref. [31], countries with a strong specialization in ICT exports have higher productivity and economic growth rates [33]. investigated the impact of ICT in 120 industrialized and developing countries, showing that a 10% increase in ICT adoption translates into an increase of 0.81% in economic growth in low- and middle-income nations. As a result [30], asserted that once a critical mass of technological infrastructure is established, ICT shows a significant causal link with economic growth in developing countries.

Additionally [34], conducted a panel data regression for 33 developing countries to determine whether higher ICT adoption leads to higher total factor productivity growth. According to this study, ICT adoption and increased educational attainment are the most significant factors positively influencing productivity in developing countries. ICT play an important role in the development of each economic sector, particularly during the liberalization process ([34]) [13]. identified a positive linear relationship between ICT and sustainable economic growth in developing countries. Specifically, this study argued that ICT is the enabler of financial inclusion, whereby underprivileged communities can access financial services, such as payment systems, investment options, and miscellaneous information.

To the best of our knowledge, most studies in the extant literature have reached the same conclusion: ICT positively contributes to economic activity ([17,20]). Despite this positive effect, there has nonetheless been a debate in the literature about the effect size on developed and developing economies ([7]). Specifically [9], argued that the effect of ICT on a country's income depends on its development level [35]. identified a higher effect of ICT on developing economies than on developed economies, while [36] claimed the opposite. A few studies, such as [37,38], did not identify a statistically significant effect of Internet use on productivity. Previously [39], introduced workforce skills to the discussion to explain the relationship between ICT and a country's level of development, arguing that workforce education is a determinant of effective ICT use with respect to a country's economic development.

The importance of a qualified workforce for economic prosperity has been documented in the literature ([[40], [41], [42]]), as well-developed human capital can use ICT more intensively ([43]). Human capital is the key to higher economic development, which is supported by socio-economic growth ([10,14,15]). According to Refs. [42,44], education is a major human capital growth driver. The positive effect of the interaction between education and ICT on sustainable economic growth has been supported by the majority of studies in the literature ([[45], [46], [47], [48]]). According to Ref. [49], this positive effect deriving from the interaction between education and technology use is found both in developed and developing economies [10]. pointed out that the technological infrastructure in high-income and upper-middle-income countries plays a significant role in education, influencing innovation processes and contributing to economic growth [50]. emphasized the role of higher education in economic development, as a well-educated workforce expects higher income and better employment prospects during their working life ([51]).

On the other hand [52], argued that well-developed human capital does not contribute to the economic growth of developed countries. Similarly, an earlier study by Ref. [39] identified a direct relationship between years of schooling and economic growth for developing countries only, which could be explained by the progressively diminishing marginal return to education because of the plethora of highly skilled workers in developed countries ([53]).

The literature has not yet thoroughly examined the impact of ICT in conjunction with R&D on productivity ([17]). Although both contribute to productivity improvement, studies like [54] argued that these two factors are inversely related. Others, such as [55], found complementarity and a direct relationship between them [56]. argued that R&D moderates the direct relationship between ICT and business performance. Irrespective of the contradictory findings regarding the relationship between ICT and R&D, most studies have highlighted the linking role of workforce skills in enabling ICT and R&D to boost organizational efficiency ([57,58]). As discussed above, education is a major driver of workforce skill development.

## 3 Methodology and data

We obtained group-specific (i.e., Group 1: developed countries; Group 2: developing countries) efficiency and meta-efficiency estimates from a Bayesian data envelopment analysis (DEA) approach. The Bayesian method used in this study has proven to yield valid estimates that perform better than other bias-corrected efficiencies yielded by counterpart techniques ([59,60]). The Bayesian DEA efficiency estimates and the corresponding technology gap are then regressed on education-, ICT-, and R&D-related variables using iterative and two-step generalized methods of moments (GMM). These linear dynamic panel data analytical approaches are regarded as appropriate because they control for reverse causality and endogeneity.

This section (see subsection 3.4) also presents the variables and the descriptive statistics of the data used to obtain empirical results.

### 3.1 Efficiencies and technology gap

We use a generalized directional distance function (GDDF) model ([61]) to measure efficiency. Given that the analysis is non-oriented and there are no undesirable variables or irregular data (e.g., negative values), the standard GDDF program reads as follows:

$$\theta^{\kappa }=\min\frac{1-\frac{1}{m}\left(\sum_{i=1}^{m}\beta^{\kappa }g_{i}/x_{io}^{\kappa }\right)}{1+\frac{1}{s}\left(\sum_{r=1}^{s}\beta^{\kappa }g_{r}/y_{ro}^{\kappa }\right)}$$

$$s.t.\;\sum_{j=1}^{n}\lambda_{j}^{\kappa }x_{ij}^{\kappa }+\beta^{\kappa }g_{x}\leq x_{io}^{\kappa }\;i=1,...,m$$

$$\sum_{j=1}^{n}\lambda_{j}^{\kappa }y_{rj}^{\kappa }-\beta^{\kappa }g_{y}\geq y_{ro}^{\kappa }\;r=1,...,s$$

$$\sum_{j=1}^{n}\lambda_{j}^{\kappa }=1$$

(1) $$\lambda_{j}^{\kappa }\geq 0,\kappa =1,...,Κ$$

$${\;g}_{x}=g_{y}=1$$

where $\theta^{\kappa }$ expresses group-specific efficiency (group κ, κ=1,...,Κ, and ${\left\{\theta_{j}^{\kappa }\right\}}_{j=1}^{n}$ ∈(0,1]), and $x_{ij}^{\kappa }$ and $y_{rj}^{\kappa }$ are the inputs (i.e., $x_{1j}^{\kappa },\ldots ,x_{mj}^{\kappa }$) and outputs (i.e., $y_{1j}^{\kappa },\ldots ,y_{sj}^{\kappa }$), respectively, of the j th unit. Also, $g_{x}$ and $g_{y}$ are the direction vectors for the inputs and outputs, respectively, and $\lambda_{j}^{\kappa }$ are the optimal weights assigned to inputs and outputs. The ratios $\beta^{\kappa }g_{i}/x_{io}^{\kappa }$ and $\beta^{\kappa }g_{r}/y_{ro}^{\kappa }$ are the proportional input decrease and output increase, respectively, for the reference unit o.

The meta-frontier framework draws on the presence of multiple technologies (i.e., κ=1,…,Κ). Hence, the meta-technology is expressed as follows:

$$T^{meta}\left(x\right)=\left\{T^{1}\left(x\right)\cup T^{2}\left(x\right)\cup \ldots \cup T^{Κ}\left(x\right)\right\}$$

where $T^{\kappa }\left(x\right)=\{y:x$ used by units in group κ can produce y}.

Therefore, the meta-efficiencies are obtained as follows:

$$\theta^{meta}=\min\frac{1-\frac{1}{m}\left(\sum_{i=1}^{m}\beta^{meta}g_{i}/x_{io}^{\kappa }\right)}{1+\frac{1}{s}\left(\sum_{r=1}^{s}\beta^{meta}g_{r}/y_{ro}^{\kappa }\right)}$$

$$s.t.\;\sum_{\kappa =1}^{Κ}\sum_{j=1}^{n}v_{j}^{\kappa }x_{ij}^{\kappa }+\beta^{meta}g_{x}\leq x_{io}^{\kappa }\;i=1,...,m$$

$$\sum_{\kappa =1}^{Κ}\sum_{j=1}^{n}v_{j}^{\kappa }y_{rj}^{\kappa }-\beta^{meta}g_{y}\geq y_{ro}^{\kappa }\;r=1,...,s$$

$$\sum_{\kappa =1}^{Κ}\sum_{j=1}^{n}v_{j}^{\kappa }=1$$

(2) $$v_{j}^{\kappa }\geq 0,\kappa =1,...,Κ$$

$${\;g}_{x}=g_{y}=1$$

where $v_{j}^{\kappa }$ are the optimal intensities.

The metafrontier framework facilitates the analysis of units' (e.g., countries’) technology heterogeneity captured by the technology gap. This gap is associated with the relative distance of a unit from the group-specific frontier to which it belongs and the metafrontier. The presence of a technology gap is attributed to (a) national market structure, (b) national regulations ([62]), (c) national institutional framework ([63]), and (d) national knowledge ecosystem ([64]). Technology gaps (TG) express inefficiency and are calculated as follows ((3) and (4)):

(3) $$TG\left(x,y\right)=\theta^{\kappa }\times \left(1-MTR\left(x,y\right)\right)$$

where MTR stands for meta-technology ratio

(4) $$MTR\left(x,y\right)=\theta^{meta}/\theta^{\kappa },0<MTR\left(x,y\right)\leq 1$$

### 3.2 Bias-corrected efficiencies

Acknowledging the upward bias of DEA efficiencies for finite samples, such as the sample used in this study ([26,59]), we introduce the efficiencies obtained from programs (1) and (2) into the bias-correction Bayesian DEA approach ([60]). This approach is based on two distributional assumptions: a uniform likelihood and a beta prior. ([65]: 325–326) proved that the mix of these two distributions yields a posterior mean resembling the parameter's estimator.

To be more precise, let ${\left\{\theta_{j}^{\kappa }\right\}}_{j=1}^{l}\in [\theta_{L}^{\kappa },1)$, where $\theta_{L}^{\kappa }\in \left(0,1\right)$ and l⊂n express the group-specific efficiencies except for the ones. Acknowledging that the maximum likelihood estimator (MLE) is asymptotically unbiased and consistent with the parameter $\theta_{L}^{\kappa }$, the expected value (5) and the unbiased estimator (6) are as follows:

(5) $$E_{l}\left\{{\hat{\theta }}_{L}^{\kappa }\right\}=\theta_{L}^{\kappa }+\frac{1-\theta_{L}^{\kappa }}{l+1}$$

and

(6) $${\tilde{\theta }}_{L}^{\kappa }=\frac{{\hat{\theta }}_{L}^{\kappa }\left(l+1\right)-1}{l}$$

Letting the parameter $\theta_{L}^{\kappa }$ be beta distributed (shape parameters: γ>0 and δ>0), the Bayesian prior reads as follows (expression (7)):

(7) $$f_{\theta_{L}^{\kappa }}\left(\theta_{L}^{\kappa }|\gamma ,\delta \right)=\frac{1}{B\left(\gamma ,\delta \right)}{\left(\theta_{L}^{\kappa }\right)}^{\gamma -1}{\left(1-\theta_{L}^{\kappa }\right)}^{\delta -1},\theta_{L}^{\kappa }\in \left(0,1\right)$$

Assuming that the unbiased estimator ${\tilde{\theta }}_{L}^{\kappa }$ (see expression (6)) is equal to the expected value of the prior distribution $\left\{\theta_{L}^{\kappa }\right\}=\frac{\gamma }{\gamma +\delta }$ , the shape parameter δ is as follows:

(8) $$\delta =\frac{\left(1-{\tilde{\theta }}_{L}^{\kappa }\right)\gamma }{{\tilde{\theta }}_{L}^{\kappa }}$$

The joint probability density function (PDF) of the vector $\Theta^{\kappa }={\left\{\theta_{j}^{\kappa }\right\}}_{j=1}^{l}$ reads as follows:

(9) $$f_{\Theta^{\kappa }}\left(\Theta^{\kappa }\right)=\int_{0}^{1}f\left(\Theta^{\kappa }|\theta_{L}^{\kappa }\right)f_{\theta_{L}^{\kappa }}\left(\theta_{L}^{\kappa }|\gamma ,\delta \right)d\theta_{L}^{\kappa }=\frac{B\left(\gamma ,\delta -l\right)}{B\left(\gamma ,\delta \right)}$$

(10) $$\text{where}\;\delta >l\;\text{and}\;\gamma >l\frac{{\tilde{\theta }}_{L}^{\kappa }}{1-{\tilde{\theta }}_{L}^{\kappa }}$$

Using the PDF of $\Theta^{\kappa }|\theta_{L}^{\kappa }$ (expression (9)), we express the Bayesian PDF of $\theta_{L}^{\kappa }|\Theta^{\kappa }$ as shown in expression (11):

(11) $$f_{\theta_{L}^{\kappa }|\Theta^{\kappa }}\left(\Theta^{\kappa }\right)=\frac{f_{\Theta^{\kappa }|\theta_{L}^{\kappa }}\left(\Theta^{\kappa }|\theta_{L}^{\kappa }\right)f_{\theta_{L}^{\kappa }}\left(\theta_{L}^{\kappa }|\gamma ,\delta \right)}{f_{\Theta^{\kappa }}\left(\Theta^{\kappa }\right)}=\frac{1}{B\left(\gamma ,\delta -l\right)}{\left(\theta_{L}^{\kappa }\right)}^{\gamma -1}{\left(1-\theta_{L}^{\kappa }\right)}^{\left(\delta -l\right)-1}$$

with shape parameters γ and δ−l.

The posterior beta distribution identifies the overestimation of group-specific efficiencies, which is shifted to the right compared to the prior distribution. In this regard, the prior distribution (expression (12)) expresses the corresponding bias-corrected efficiencies.

(12) $${\;E}_{l}\left\{\theta_{L}^{\kappa }|\Theta^{\kappa }\right\}>E_{l}\left\{\theta_{L}^{\kappa }\right\}\;\text{as}\;\frac{\gamma }{\gamma +\delta -l}>\frac{\gamma }{\gamma +\delta }$$

where δ>l (see the constraints in (10)) prevents problems with the posterior (i.e., $E_{l}\left\{\theta_{L}^{\kappa }|\Theta^{\kappa }\right\}↛0$).

Based on expression (6), efficiencies are corrected from bias as follows:

(13) $$\xi =\frac{{\tilde{\theta }}_{L}^{\kappa }}{{\hat{\theta }}_{L}^{\kappa }}<1,\text{where}\;\text{the}\;\text{MLE}\;{\hat{\theta }}_{L}^{\kappa }=\min\;\Theta^{\kappa }$$

Using expressions (8), (10), and (13), we obtain (14) and (15)

(14) $$\hat{\gamma }=l{\tilde{\theta }}_{L}^{\kappa }/\left(1-\xi \right)$$

and

(15) $$\hat{\delta }=\left(1-{\tilde{\theta }}_{L}^{\kappa }\right)\hat{\gamma }/{\tilde{\theta }}_{L}^{\kappa }$$

(14) and (15) are not user-defined but estimated using the MATLAB function *betarnd*.

To obtain the bias-corrected efficiencies ${\left(\theta_{j}^{\kappa }\right)}^{c}$, we fit the prior beta distribution to the posterior beta distribution ratio with a normal distribution using the MATLAB function *normfit* with parameters $\hat{\mu }$ and $\hat{\sigma }$.

(16) $${\;\left(\theta_{j}^{\kappa }\right)}^{c}=w^{-1}\sum_{\rho =1}^{w}{\overset{˘}{\theta }}_{j\rho }^{\kappa }$$

where w expresses Monte Carlo iterations (ρ=1,000), and ${\overset{˘}{\theta }}_{j\rho }^{\kappa }$ are randomly generated efficiencies obtained from the MATLAB function *normrnd* with parameters $\theta_{j}^{\kappa }\hat{\mu }$ and $\theta_{j}^{\kappa }\hat{\sigma }$ ($\theta_{j}^{\kappa }$ are obtained from program (1)).

The same Bayesian bias-correction procedure presented in expressions (5)–(16) applies to meta-efficiencies ($\theta_{j}^{meta}$), yielding ${\left(\theta_{j}^{meta}\right)}^{c}$. Then, the technology gap and the meta-technology ratio are estimated as shown in expressions (17) and (18):

(17) $${TG}^{c}\left(x,y\right)={\left(\theta^{\kappa }\right)}^{c}\times \left(1-{MTR}^{c}\left(x,y\right)\right)$$

(18) $${MTR}^{c}\left(x,y\right)={\left(\theta^{meta}\right)}^{c}/{\left(\theta^{\kappa }\right)}^{c},0<{MTR}^{c}\left(x,y\right)\leq 1$$

### 3.3 GMM estimates

To estimate the impact of education, ICT, and R&D on economic efficiency and the technology gap (TG), we use a two-step and iterative GMM for linear dynamic panel data. GMM can address possible endogeneity and feedback effects between independent and dependent variables (i.e., economic efficiency and TG) ([66]). Feedback effects are relaxed in the regression model by using economic efficiency and TG as response variables, obtained from the Bayesian DEA approach discussed in Section 3.2, instead of conventional proxies to express economic growth, such as GDP or GDP per capita. As a result, the distributions of the Bayesian DEA economic efficiency and TG lie at (0,1) and [0,0.152] intervals, respectively.

Drawing on the panel linear dynamic regression models found in Refs. [67,68], we reach the following expressions (i.e., expressions (19)–(22) refer to economic efficiency, while expressions (23)–(25) refer to TG). Expressions (19)–(25) take into account aggregate effects influencing individual panel observations. All of the following expressions satisfy specification, overidentification, and linearity tests. Details about these tests (i.e., Arellano and Bond test, Hansen *J*-test, and Wald test) are available in Section 5. It goes without saying that models failing to satisfy the above tests are not presented in this study.

(19) $$\theta_{j,t}^{c}=a{\bar{\theta }}_{j,t-1}^{c}+b_{1}{\bar{z}}_{1,j,t-1}+b_{2}{\bar{z}}_{2,j,t-2}+b_{3}{\bar{z}}_{5,j,t-2}+\varphi_{p+1}d_{p+1}+\ldots +\varphi_{T}d_{T}+\eta_{j}+\varepsilon_{j,t},j=1,\ldots ,n;\;t=p+1,\ldots ,T;\;p=1,2$$

(20) $$\theta_{j,t}^{c}=a{\bar{\theta }}_{j,t-1}^{c}+b_{1}{\bar{z}}_{1,j,t-1}+b_{2}{\bar{z}}_{2,j,t-2}\;{+b}_{3}{\bar{z}}_{3,j,t-2}+b_{4}{\bar{z}}_{4,j,t-2}+{\tau I}_{j,t}+\varphi_{p+1}d_{p+1}+\ldots +\varphi_{T}d_{T}+\eta_{j}+\varepsilon_{j,t},j=1,\ldots ,n;\;t=p+1,\ldots ,T;p=1,2$$

(21) $$\theta_{j,t}^{c}=a{\bar{\theta }}_{j,t-1}^{c}+b_{1}{\bar{z}}_{1,j,t-1}+{b_{2}\bar{z}}_{4,j,t-2}+b_{3}{\bar{z}}_{5,j,t-2}+b_{4}{\bar{z}}_{1,j,t-1}*{\bar{z}}_{5,j,t-2}+\varphi_{p+1}d_{p+1}+\ldots +\varphi_{T}d_{T}+\eta_{j}+\varepsilon_{j,t},j=1,\ldots ,n;\;t=p+1,\ldots ,T;\;p=1,2$$

(22) $$\theta_{j,t}^{c}=a{\bar{\theta }}_{j,t-1}^{c}+b_{1}{\bar{z}}_{1,j,t-1}+b_{2}{\bar{z}}_{2,j,t-2}+b_{3}{\bar{z}}_{3,j,t-2}+b_{4}{\bar{z}}_{4,j,t-2}+b_{5}{\bar{z}}_{1,j,t-1}*{\bar{z}}_{2,j,t-2}+\varphi_{p+1}d_{p+1}+\ldots +\varphi_{T}d_{T}+\eta_{j}+\varepsilon_{j,t},j=1,\ldots ,n;\;t=p+1,\ldots ,T;\;p=1,2$$

where the response variable (i.e., $\theta_{j,t}^{c}$) is the bias-corrected efficiency (expression (16)) assigned to each sample country (j=1,…,n) during the review period (t=p+1,…,T;p=1,2), and ${\bar{\theta }}_{j,t-1}^{c}$ expresses the lagged bias-corrected efficiency at the aggregate level. The lagged independent variables are denoted by ${\bar{z}}_{1,j,t-1}$, ${\bar{z}}_{2,j,t-2}$, ${\bar{z}}_{3,j,t-2}$, ${\bar{z}}_{4,j,t-2}$, and ${\bar{z}}_{5,j,t-2}$. In models (19)–(22), we estimate their aggregate effects on bias-corrected economic efficiency. $I_{j,t}$ is a dummy variable that represents the j th country classification as developed (code 0) or developing (code 1). The variables $d_{p+1},\ldots ,d_{T}$ are time dummies with their respective coefficients $\varphi_{p+1},\ldots ,\varphi_{T}$. The unobserved individual-specific effect is expressed by η, while ε is the idiosyncratic remainder component. The lagged response coefficient a along with $b_{1},b_{2},b_{3},b_{4}$ and $b_{5}$ are the average fixed-effect coefficients. The coefficient τ is the average country-specific impact of a country classified as developing instead of developed on economic efficiency at the current moment.

(23) $${TG}_{j,t}^{c}=a{\bar{TG}}_{j,t-1}^{c}+b_{1}{\bar{z}}_{1,j,t-1}+b_{2}{\bar{z}}_{4,j,t-2}+b_{3}{\bar{z}}_{5,j,t-2}+\varphi_{p+1}d_{p+1}+\ldots +\varphi_{T}d_{T}+\eta_{j}+\varepsilon_{j,t},j=1,\ldots ,n;\;t=p+1,\ldots ,T;\;p=1,2$$

(24) $${TG}_{j,t}^{c}=a{\bar{TG}}_{j,t-1}^{c}+b_{1}{\bar{z}}_{1,j,t-1}+b_{2}{\bar{z}}_{4,j,t-2}+b_{3}{\bar{z}}_{5,j,t-2}+b_{4}{\bar{z}}_{1,j,t-1}*{\bar{z}}_{4,j,t-2}+\varphi_{p+1}d_{p+1}+\ldots +\varphi_{T}d_{T}+\eta_{j}+\varepsilon_{j,t},j=1,\ldots ,n;\;t=p+1,\ldots ,T;\;p=1,2$$

(25) $${TG}_{j,t}^{c}=a{\bar{TG}}_{j,t-1}^{c}+b_{1}{\bar{z}}_{1,j,t-1}+b_{2}{\bar{z}}_{4,j,t-2}\;{+b}_{3}{\bar{z}}_{5,j,t-2}+\tau I_{j,t}+\varphi_{p+1}d_{p+1}+\ldots +\varphi_{T}d_{T}+\eta_{j}+\varepsilon_{j,t},j=1,\ldots ,n;\;t=p+1,\ldots ,T;\;p=1,2$$

where ${TG}_{j,t}^{c}$ is the bias-corrected technology gap and is obtained from expression (17). Also, ${\bar{TG}}_{j,t-1}^{c}$ represents the lagged bias-corrected technology gap at the aggregate level.

### 3.4 Sample and data Description

The sample used in this study consists of 52 countries, classified as developed (31 developed countries) or developing (21 developing countries). The list of sample countries is available in Table A1 in the Appendix. The selection of these countries was solely based on the availability of data for all variables incorporated in efficiency estimation and regression analysis throughout the review period (2011–2019); hence, the data analysis drew on a balanced panel. The classification of the sample countries into developed or developing was based on the IMF World Economic Outlook database (https://www.imf.org/en/Publications/WEO/weo-database/2022/April/select-aggr-data).

For estimating economic efficiency, we used the standard economic variables—i.e., inputs: labor (in millions) and capital stock (in millions of $), and output: GDP (in millions of $) ([69]). Emphasizing the independent variables introduced into the regression models (i.e., expressions (19)–(25)), the ICT proxies were (1) fixed broadband subscriptions per 100 people ($z_{2}$), (2) individuals using the Internet (% of the population) ($z_{3}$), and (3) mobile cellular subscriptions per 100 people ($z_{4}$). These ICT proxies are widely used in the literature; for instance, fixed broadband subscriptions are used in the studies of [39,70], and [71], while individuals using the Internet were found in the papers of [19,39], and [72], and the mobile subscriptions variable was used by Refs. [19,39], and [73].

The education expenditure (% of Gross National Income – GNI) ($z_{1}$) served as the education proxy in this study's regression models. Various alternative education variables have been used in the literature, such as the percentage of employees with tertiary-level education ([74]), educational attainment ([75]), and years of education ([70]), and the percentage of the gross enrolment ratio for both sexes ([19]). In this study, we collected data for various educational variables in addition to the education expenditure proxy, such as (1) compulsory education expenditure (years); (2) current education expenditure, tertiary; (3) current education expenditure, total; (4) educational attainment, at least bachelor's or equivalent; (5) educational attainment, at least completed; (6) educational attainment, at least master's or equivalent; (7) educational attainment, doctoral or equivalent; (8) expenditure on tertiary education (% of government expenditure on education); (9) government expenditure on education, total (% of GDP); (10) labor force with advanced education (% of total working-age population with advanced education); (11) labor force with basic education (% of working-age population with basic education); and (12) labor force with intermediate education (% of total working-age population with intermediate education). However, none of the above alternative educational variables were used in the analysis either because of the large number of missing values, which would considerably reduce the sample size and the review period, or the inappropriateness of those variables for the regression models and the GMM diagnostics, which could not be satisfied.

The innovation proxy used in this study was R&D expenditure (% of GDP) ($z_{5}$). The same variable was also used by Ref. [30] to express innovation, as well as R&D personnel (full-time equivalent) and the number of authorized patent applications [17]. incorporated firm-level expenditure on R&D to assess innovation and productivity.

Drawing on a correlation analysis (see Table A2 in the Appendix) of all the variables above, R&D expenditure ($z_{5}$) is strongly and significantly correlated with fixed broadband subscriptions and individuals using the Internet. Unexpectedly, a strong inverse relationship, which is statistically significant, is found between education expenditure ($z_{1}$) and fixed broadband subscriptions and individuals using the Internet. Also, a moderate negative relationship is reported between education expenditure and R&D expenditure, which is statistically significant only for developed countries. The same finding for developed economies has been raised by Ref. [53]. According to the World Bank, education expenditure refers to operating costs for education, including wages and salaries. The education expenditure variable calculation excludes investments in buildings or equipment associated with education. Also, the R&D expenditure covers basic and applied research as well as experimental development. Specifically, a perfect positive and significant relationship between the individuals using the Internet ($z_{3}$) and fixed broadband subscriptions ($z_{2}$) is present.

In addition to the ICT, education, and R&D proxies used in this study, we also introduced control variables into the regression models. Furthering [19,76] studies, we incorporated (1) trade (% of GDP), expressing the openness of an economy, and (2) consumer price index (annual change %) in conjunction with the main explanatory variables in GMM. None of these control variables had a statistically significant impact on economic efficiency, while all regression models incorporating them and combinations of the main explanatory variables did not satisfy the diagnostic tests (i.e., Arellano and Bond test, Hansen *J*-test, and Wald test).

The data for all variables above are available in the World Bank database (i.e., World Development Indicators: https://databank.worldbank.org/source/world-development-indicators).

**Fig. 1:**
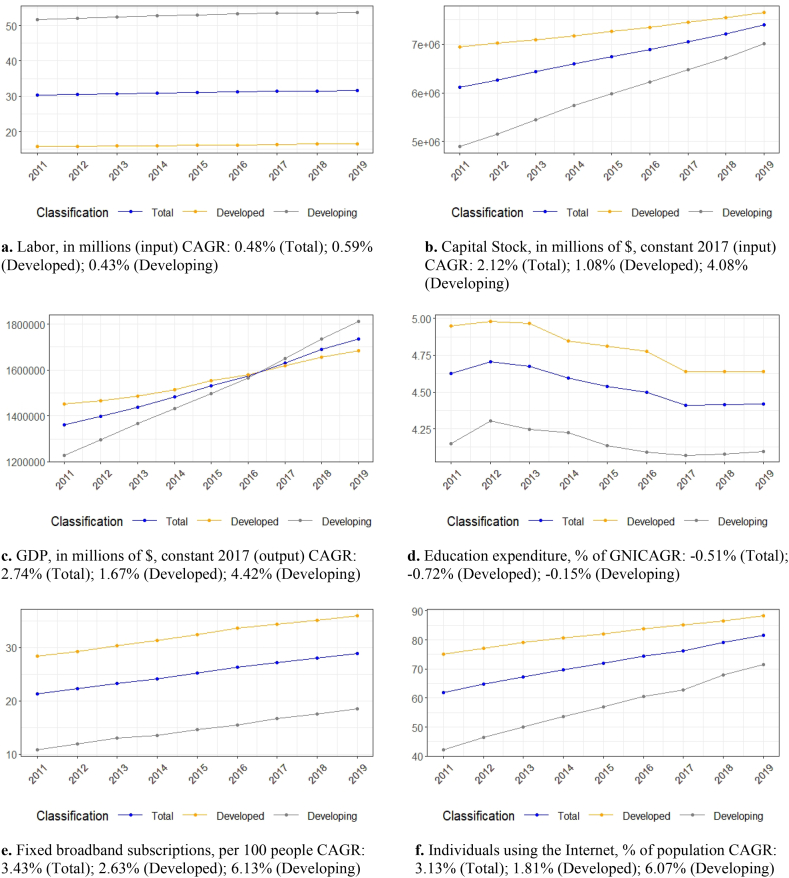

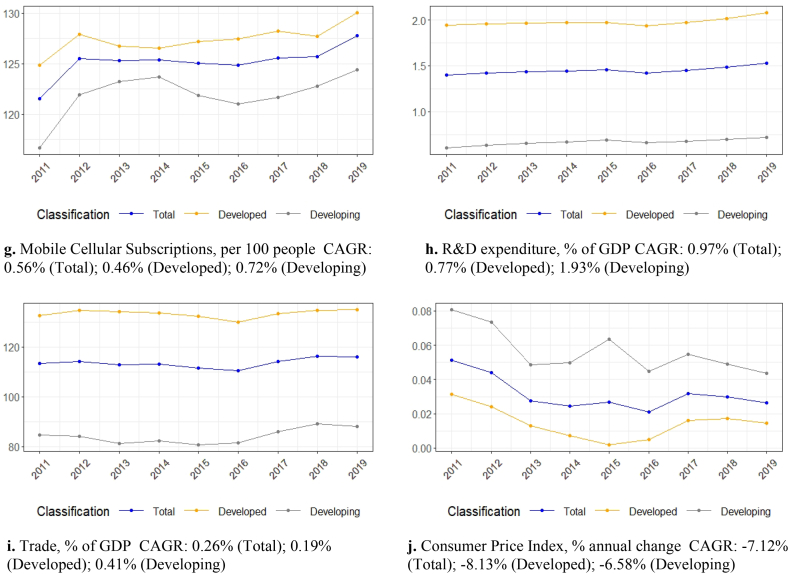
Data trends, Fig. 1a. Labor, in millions (input), CAGR: 0.48% (Total); 0.59% (Developed); 0.43% (Developing), Fig. 1b. Capital Stock, in millions of $, constant 2017 (input), CAGR: 2.12% (Total); 1.08% (Developed); 4.08% (Developing), Fig. 1c. GDP, in millions of $, constant 2017 (output), CAGR: 2.74% (Total); 1.67% (Developed); 4.42% (Developing), Fig. 1d. Education expenditure, % of GNI, CAGR: −0.51% (Total); −0.72% (Developed); −0.15% (Developing), Fig. 1e. Fixed broadband subscriptions, per 100 people, CAGR: 3.43% (Total); 2.63% (Developed); 6.13% (Developing), Fig. 1f. Individuals using the Internet, % of population, CAGR: 3.13% (Total); 1.81% (Developed); 6.07% (Developing), Fig. 1g. Mobile Cellular Subscriptions, per 100 people, CAGR: 0.56% (Total); 0.46% (Developed); 0.72% (Developing), Fig. 1h. R&D expenditure, % of GDP, CAGR: 0.97% (Total); 0.77% (Developed); 1.93% (Developing), Fig. 1i. Trade, % of GDP, CAGR: 0.26% (Total); 0.19% (Developed); 0.41% (Developing), Fig. 1j. Consumer Price Index, % annual change, CAGR: −7.12% (Total); −8.13% (Developed); −6.58% (Developing)

On the one hand, as expected, the workforce in developing countries was considerably higher than in developed countries between 2011 and 2019 (Fig. 1a). On the other hand, the capital stock of the latter group was greater than the capital stock of the developing countries. As a result, the figures converged during the review period, especially after 2015 (Fig. 1b). It is noteworthy that the average GDP of the sample developing countries exceeded the average GDP of the sample developed countries after 2016 (Fig. 1c). Regarding the proxy variables, education expenditure decreased from 2011 to 2019 for both developed and developing countries (Fig. 1d). The education spending cuts were more significant for the sample developed countries (compound annual growth rate (CAGR): −0.72%). According to the World Bank, education expenditure refers to educational operating costs, including wages and salaries. The education expenditure calculation excludes investments in buildings and equipment associated with education. Unlike education expenditure, R&D spending rose from 2011 to 2019 (Fig. 1h). Drawing on the World Bank's definition, R&D expenditure covers basic and applied research, as well as experimental development. Like R&D expenditure, all ICT figures increased considerably during the review period (Fig. 1e, f, and 1g). The average growth rates for the ICT proxies were stronger for the sample developing countries compared to the sample developed countries, which is an expected finding. As shown in Section 4, ICT plays a critical role in knowledge dissemination and technology heterogeneity convergence between developed and developing countries. Finally, trade increased between 2011 and 2019, mainly for the developing countries (Fig. 1i), while the consumer price index significantly decreased for both groups of sample countries (Fig. 1j).

## 4 Empirical results

**Fig. 2:**
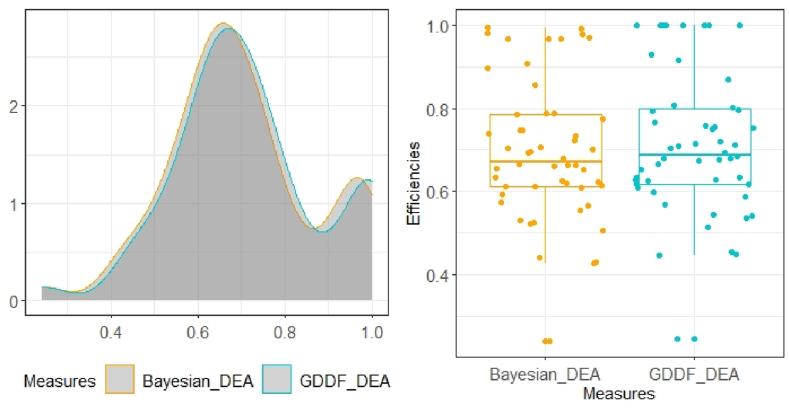
Efficiency densities.

Expressions (1)–(16) yield efficiency estimates (i.e., group-specific efficiencies and meta-efficiencies) as well as technology gap inefficiency estimates (expressions (17) and (18)). The bias correction performed by the Bayesian DEA approach discussed in Section 3.2 is illustrated in Fig. 2, where the density of the Bayesian DEA estimates is shifted to the left and up compared to that of the DEA scores obtained from programs (1) or (2). Also, as shown in the boxplots in the same graph, there are no ones.

Sample countries' economic efficiency improved from 2012 to 2019. Specifically, the developed countries' efficiency presented steady growth between 2012 and 2019, while developing countries’ efficiency showed strong growth after 2015. Overall, developed countries presented, on average, higher efficiency than sample developing countries, as well as a higher compound annual growth rate (CAGR) during the review period (2011–2019) of 0.83%, compared to the CAGR of their developing counterparts (0.61%).

**Fig. 3:**
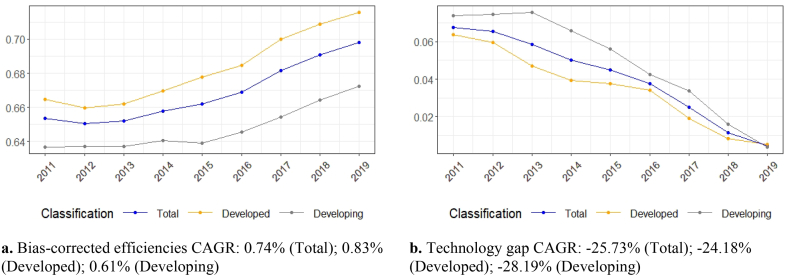
Efficiencies and technology gaps Fig. 3a. Bias-corrected efficiencies, CAGR: 0.74% (Total); 0.83% (Developed); 0.61% (Developing), Fig. 3b. Technology gap,CAGR: −25.73% (Total); −24.18% (Developed); −28.19% (Developing).

The strong growth of developing countries' efficiency after 2015 has led to the convergence of technology gap inefficiencies between the sample's developed and developing countries (Fig. 3). This is a noteworthy finding because it reveals the gradual lift of barriers to economic efficiency expansion associated with factors such as a country's market structure and regulatory framework, the country-level economic and knowledge ecosystem, and the country's culture. Specifically, developing countries' technology gap inefficiency declined by 28.19% on average, while that of the developed countries declined by 24.18% in the 2011–2019 period.

Table 1 includes only estimates obtained from models that satisfy specification (Arellano and Bond test), overidentification (Hansen *J*-test), and linearity (Wald test) tests. The estimates are assigned to cross-section aggregate variables, expressing aggregate effects.

**Table 1:** Impact of Education, ICT, and R&D on Efficiency (two-step and iterative GMM estimates).

Variables	Coefficients (Models)			
	(19)	(20)	(21)	(22)
${\bar{\theta }}_{j,t-1}^{c}$	0.3256***	0.3544***	0.3178***	0.3354***
	(0.0147)	(0.0006)	(0.0149)	(0.0004)
${\bar{z}}_{1,j,t-1}$	0.0007	−0.0001	0.0023	0.0001***
	(0.007)	(0.0061)	(0.0055)	(0.0006)
${\bar{z}}_{2,j,t-2}$	0.0005*	0.0001*		0.0006*
	(0.0016)	(0.0019)		(0.0015)
				
${\bar{z}}_{3,j,t-2}$		−0.0001		0.0000
		(0.0004)		(0.0004)
${\bar{z}}_{4,j,t-2}$		0.0000	0.0000	0.0000
		(0.0003)	(0.0002)	(0.0002)
				
${\bar{z}}_{5,j,t-2}$	0.0000		0.0570***	
	(0.024)		(0.011)	
$I_{j,t}$^a^		−0.0097**		
		(0.003)		
				
${\bar{z}}_{1,j,t-1}*{\bar{z}}_{5,j,t-2}$			−0.0009	
			(0.0062)	
${\bar{z}}_{1,j,t-1}*{\bar{z}}_{2,j,t-2}$				0.0000
				(0.0002)
Year dummies	Yes	Yes	Yes	Yes
Observations	468	468	468	468
GMM	Two-step	Two-step	Iterative	Two-step
F-Statistic	4.12E+09	1.48E+10	2.45E+08	3.3E+11
p-value	<10^−3^	<10^−3^	<10^−3^	<10^−3^
Arellano and Bond test	1.2323	0.2385	−1.5970	−1.9058
p-value	0.2178	0.8115	0.1103	0.0567
Hansen *J*-test	17.10	10.80	13.61	17.69
p-value	0.9899	1.0000	0.9997	0.9987
Wald test	173781	944603	461453	1376854
p-value	<10^−4^	<10^−4^	<10^−4^	<10^−4^

Coefficients are significant at the 0.05 level (denoted by *), 0.01 level (denoted by **), and 0.0001 level (denoted by ***).

Values in parentheses are standard errors.

a Classification ($I_{j,t}$) is a dummy-coded variable, where 0 expresses developed countries and 1 is assigned to developing countries.

In line with the literature (e.g., Refs. [5,13,18]), education, ICT, and R&D positively affect economic efficiency (Table 1). Emphasizing ICT, we find that fixed broadband subscriptions (${\bar{z}}_{2,j,t-2}$) have a statistically significant positive effect on efficiency in all models that incorporate this variable (i.e., models (19), (20), and (22)). Acknowledging that the fixed broadband subscriptions variable ($z_{2}$) is perfectly positively correlated with the individuals using the Internet variable ($z_{3}$), and positively strongly correlated with the R&D expenditure variable ($z_{5}$) (Table A2 in the Appendix), it is clear why the two latter variables do not significantly impact economic efficiency when fixed broadband subscriptions are incorporated into the same model. In support of this argument, in the model (21) (see Table 1), R&D expenditure (${\bar{z}}_{5,j,t-2}$) presents a significant positive effect on efficiency, while fixed broadband subscriptions (${\bar{z}}_{2,j,t-2}$) and individuals using the Internet (${\bar{z}}_{3,j,t-2}$) were not included in the analysis.

In model (22), education (${\bar{z}}_{1,j,t-1}$) shows a significant positive impact on efficiency, as well as the number of fixed broadband subscriptions (${\bar{z}}_{2,j,t-2}$). However, given the sample of countries and variables used in this study, the interaction between these variables does not have a statistically significant effect on efficiency, as [20] found. Similarly, we could not support complementarity between education (${\bar{z}}_{1,j,t-1}$) and R&D (${\bar{z}}_{5,j,t-2}$) as identified by Refs. [14,15]. As mentioned above, when R&D (${\bar{z}}_{5,j,t-2}$) was tested together with education (${\bar{z}}_{1,j,t-1}$) (model (21)), the latter variable did not significantly affect economic efficiency. The estimates in Table 1, specifically model (20), support the findings of [7,9], and [77]. To be more precise, a country's economic development level significantly affects its economic efficiency, which is negative for developing countries (i.e., $I_{j,t}$ - Classification code 1: developing countries). In the same model (i.e., model (20)), the fixed broadband subscriptions variable (${\bar{z}}_{2,j,t-2}$) has a significant positive effect on economic efficiency.

In Table 2, the technology gap (TG) inefficiency (${TG}_{j,t}^{c}$) is regressed on education, ICT, and R&D proxies. The reduction in TG inefficiency is crucial for countries’ economic efficiency improvement, as it is associated with regulatory barriers and the framework within which countries transform their resources into GDP, but not with the production process (e.g., the transformation of inputs into outputs) per se.

**Table 2:** Impact of Education, ICT, and R&D on Technology Gap Inefficiency (two-step and iterative GMM estimates).

Variables	Coefficients	Variables	Coefficients	Variables	Coefficients
	Model (23)		Model (24)		Model (25)
${\bar{TG}}_{j,t-1}^{c}$	0.4211***	${\bar{TG}}_{j,t-1}^{c}$	0.4019***	${\bar{TG}}_{j,t-1}^{c}$	0.1028***
	(0.0006)		(0.0006)		(0.0003)
${\bar{z}}_{1,j,t-1}$	0.0002	${\bar{z}}_{1,j,t-1}$	0.0004***	${\bar{z}}_{1,j,t-1}$	0.0004
	(0.0025)		(0.0051)		(0.0056)
${\bar{z}}_{4,j,t-2}$	0.0000	${\bar{z}}_{4,j,t-2}$	−0.0002***	${\bar{z}}_{4,j,t-2}$	0.0000
	(0.0002)		(0.0018)		(0.0002)
${\bar{z}}_{5,j,t-2}$	−0.0001*	${\bar{z}}_{5,j,t-2}$	−0.0029***	${\bar{z}}_{5,j,t-2}$	−0.0044***
	(0.0043)		(0.0024)		(0.001)
		${\bar{z}}_{1,j,t-1}*{\bar{z}}_{4,j,t-2}$	−0.0001***	$I_{j,t}$^a^	0.0073***
			(0.0002)		(0.0014)
Year dummies	Yes		Yes		Yes
Observations	468		468		468
GMM	Two-step		Iterative		Iterative
F-Statistic	3.5E+10		71687508		8.16E+09
p-value	<10^−3^		<10^−3^		<10^−3^
Arellano and Bond test	−0.1971		−0.0424		−0.2827
p-value	0.8438		0.9662		0.7774
Hansen *J*-test	18.2080		2.2401		8.0637
p-value	0.9959		1.0000		1.0000
Wald test	12957		35.47		1236.1
p-value	<10^−4^		0.0123		<10^−4^

Coefficients are significant at the 0.05 level (denoted by *), 0.01 level (denoted by **), and by 0.0001 level (denoted by ***).

Values in parentheses are standard errors.

a Classification ($I_{j,t}$) is a dummy-coded variable, where 0 expresses developed countries and 1 is assigned to developing countries.

As with Table 1, only estimates obtained from models satisfying specification, overidentification, and linearity tests are illustrated in Table 2.

Apart from the lagged TG inefficiency (${\bar{TG}}_{j,t-1}^{c}$), which significantly positively affects current TG inefficiency, R&D (${\bar{z}}_{5,j,t-2}$) presents a significantly negative impact on TG in all models in Table 2. In addition to R&D, an ICT proxy (i.e., mobile cellular subscriptions (${\bar{z}}_{4,j,t-2}$)) is regarded as a driver for reducing TG inefficiency (model (24)). The economic development level (i.e., $I_{j,t}$ - Classification) also impacts TG inefficiency significantly. However, it increases TG inefficiency for developing countries (Classification code 1) compared to developed countries (Classification code 0).

All models in Table 2 (i.e., (23), (24), and (25)) assign the education proxy (${\bar{z}}_{1,j,t-1}$) positive coefficients, and according to model (24), it has a significant positive impact on TG inefficiency. This unexpected result is likely because of the higher education expenditure of developed countries compared to developing ones (Fig. 1d), which supports group-specific heterogeneity. However, ICT (i.e., mobile cellular subscriptions (${\bar{z}}_{4,j,t-2}$)) and the interaction between education and ICT (${\bar{z}}_{1,j,t-1}*{\bar{z}}_{4,j,t-2}$), which was interpreted by Ref. [20] as more educated people using ICT more effectively, almost neutralizing the significantly positive effect of the education proxy.

Comparing models (23) and (24), we find that the interaction between education and ICT strengthens the significant negative impact of R&D on TG inefficiency, which is explained by the strengthening of knowledge dissemination within and across country groups (i.e., across developed and developing countries) through R&D cooperation. According to ([78]: 127–152), education should emphasize the development of technological capabilities to enhance the impact of ICT on knowledge spillovers [77]. reached the same conclusion, drawing on a sample of 265 cities in China. Specifically, they highlighted the impact of technological innovation and penetration on technology heterogeneity reduction. In an earlier study [6], highlighted the importance of ICT in closing the efficiency gap between developed and developing countries. However, based on our findings, knowledge spillover effects facilitated by ICT penetration are responsible not only for developed and developing countries’ efficiency convergence but also for the decrease in technology heterogeneity between these two country groups.

## 5 Conclusions and policy implications

This study shed light on the contribution of education, ICT, and R&D to economic efficiency improvement and technology gap inefficiency reduction. A balanced panel of 52 countries (31 developed and 21 developing) was used during 2011–2019. Efficiencies and technology gaps were estimated by a Bayesian data envelopment analysis (DEA) method. It has been proven that this novel approach yields valid estimates, which are then regressed on education, ICT, and R&D proxy variables. Incorporating efficiency and technology gap inefficiency estimates in the regression analysis as response variables instead of GDP or GDP per capita, we mitigated possible feedback effects between the explanatory and response variables (e.g., GDP and R&D expenditure, measured as % of GDP). The regression analyses drew on two-step and iterative GMM for linear dynamic panel data. The novelty of this study was not limited to using a Bayesian DEA approach to estimate efficiencies, but, to the best of our knowledge, this is the first study to investigate the impact of education, ICT, and R&D on technology gap inefficiency estimates. In other words, to explore how the abovementioned variables affect countries’ market structures, regulatory frameworks, economic and knowledge ecosystems, and cultures.

According to our findings, while the economic efficiency of developed and developing countries grew during the review period (i.e., developed countries' compound efficiency annual growth rate was higher than that of their developing counterparts), the technology gap inefficiency of those two groups converged. This outcome showed a gradual lifting of barriers to countries' economic efficiency improvement because of environmental factors (e.g., market structure). Emphasizing economic efficiency, fixed broadband subscriptions made the most significant contribution. It should be noted, however, that this ICT proxy was perfectly positively correlated with the individuals using the Internet and strongly positively correlated with R&D expenditure. In addition to fixed broadband subscriptions, education significantly impacted efficiency. When the former variable was not incorporated into the regression analysis, R&D showed a significant positive effect on efficiency. Also, a country's economic development (classification) played a significant role in economic efficiency, which was negative for developing countries compared with developed ones. These findings were in line with the extant literature.

With respect to technology gap inefficiency, R&D has a significantly negative effect across the regression models, satisfying the specification, overidentification, and linearity tests. Also, education and ICT (i.e., mobile cellular subscriptions) strengthened the significant negative impact of R&D on technology gap inefficiency. Intuitively, we conclude that knowledge spillovers within and across country groups through R&D cooperation played a significant positive role in closing the technology gap. In addition, ICT moderates the relationship between education, R&D economic efficiency, and technology gap inefficiency.

Considering this study's findings, ICT investments should be included in national development programs. Developing countries especially need to emphasize digitalization to catch up with developed countries' economic growth. This conclusion is consistent with the catch-up literature. Also, developing countries should invest in education, particularly in advancing technological capabilities. Education is one of the three pillars of the Human Development Index (HDI), which is one of the criteria for classifying countries as developed or developing. Education investments can neutralize the country-classification effect on economic efficiency and technology gap inefficiency.

A limitation of this study is the small number of sample countries. Given that the criterion for selecting sample countries was the availability of data for all variables throughout the review period, this limitation could be addressed in the near future once additional data are available. Also, the review period could be expanded to include information covering the COVID-19 pandemic time as soon as that data becomes available. We acknowledge that our analysis did not include the standard control variables found in the relevant literature, such as trade and consumer price index. Although these control variables were initially incorporated in GMM while testing alternative combinations of variables, no model including these control variables satisfied all of the specification, overidentification, and linearity tests. In the future, we could incorporate institutional factors (e.g., rule of law, control of corruption, regulatory quality, etc.) into the analysis in addition to education, ICT, and R&D proxy variables.

## Data availability statement

The data for all variables used in this paper are available in the World Bank database (i.e., World Development Indicators: https://databank.worldbank.org/source/world-development-indicators).

## CRediT authorship contribution statement

**Rashid S. Aljneibi:** Validation, Investigation, Formal analysis, Data curation, Writing – original draft. **Panagiotis D. Zervopoulos:** Writing – review & editing, Visualization, Validation, Supervision, Software, Methodology, Formal analysis, Conceptualization, Writing – original draft. **Angelos Kanas:** Conceptualization, Resources, Writing – review & editing.

## Declaration of competing interest

The authors declare that they have no known competing financial interests or personal relationships that could have appeared to influence the work reported in this paper.

## Figures and Tables

**Table A1:** Sample countries

Developed countries						Developing countries		
#	Name	Code	#	Name	Code	#	Name	Code
1	Austria	AUT	22	Latvia	LVA	1	Armenia	ARM
2	Belgium	BEL	23	Malta	MLT	2	Bulgaria	BGR
3	Canada	CAN	24	Netherlands	NLD	3	Belarus	BLR
4	Cyprus	CYP	25	Norway	NOR	4	Chile	CHL
5	Czech Republic	CZE	26	Portugal	PRT	5	China	CHN
6	Germany	DEU	27	Singapore	SGP	6	Colombia	COL
7	Denmark	DNK	28	Slovak Republic	SVK	7	Egypt, Arab Rep.	EGY
8	Spain	ESP	29	Slovenia	SVN	8	Croatia	HRV
9	Estonia	EST	30	Sweden	SWE	9	Hungary	HUN
10	Finland	FIN	31	United States	USA	10	Kazakhstan	KAZ
11	France	FRA				11	Mexico	MEX
12	United Kingdom	GBR				12	North Macedonia	MKD
13	Greece	GRC				13	Mongolia	MNG
14	Hong Kong SAR, China	HKG				14	Poland	POL
15	Ireland	IRL				15	Russian Federation	RUS
16	Israel	ISR				16	El Salvador	SLV
17	Italy	ITA				17	Serbia	SRB
18	Japan	JPN				18	Tunisia	TUN
19	Korea, Rep.	KOR				19	Turkey	TUR
20	Lithuania	LTU				20	Ukraine	UKR
21	Luxembourg	LUX				21	South Africa	ZAF

**Table A2:** Correlations (main explanatory variables)

Variables	Education expenditure [$z_{1}$] (% of GNI)	Fixed broadband subscriptions [$z_{2}$] (per 100 people)	Individuals using the Internet [$z_{3}$] (% of population)	Mobile cellular subscriptions [$z_{4}$] (per 100 people)	R&D expenditure [$z_{5}$] (% of GDP)
All countries					
Education expenditure [$z_{1}$] (% of GNI)	1				
Fixed broadband subscriptions [$z_{2}$] (per 100 people)	−0.883∗∗	1			
Individuals using the Internet [$z_{3}$] (% of population)	−0.883∗∗	1.000∗∗	1		
Mobile cellular subscriptions [$z_{4}$] (per 100 people)	−0.483	0.683∗	0.683∗	1	
R&D expenditure [$z_{5}$] (% of GDP)	−0.617	0.800∗∗	0.800∗∗	0.783∗	1
Developed countries					
Education expenditure [$z_{1}$] (% of GNI)	1				
Fixed broadband subscriptions [$z_{2}$] (per 100 people)	−0.932∗∗	1			
Individuals using the Internet [$z_{3}$] (% of population)	−0.932∗∗	1.000∗∗	1		
Mobile cellular subscriptions [$z_{4}$] (per 100 people)	−0.559	0.700∗	0.700∗	1	
R&D expenditure [$z_{5}$] (% of GDP)	−0.678∗	0.750∗	0.750∗	0.567	1
Developing countries					
Education expenditure [$z_{1}$] (% of GNI)	1				
Fixed broadband subscriptions [$z_{2}$] (per 100 people)	−0.783∗	1			
Individuals using the Internet [$z_{3}$] (% of population)	−0.783∗	1.000∗∗	1		
Mobile cellular subscriptions [$z_{4}$] (per 100 people)	0.25	0.35	0.35	1	
R&D expenditure [$z_{5}$] (% of GDP)	−0.617	0.917∗∗	0.917∗∗	0.5	1

∗ Correlation is significant at the 0.05 level (2-tailed).

∗∗ Correlation is significant at the 0.01 level (2-tailed).

## References

[1] Acemoglu D., Robinson J.A. (2013).

[2] Benos N., Zotou S. (2014). Education and economic growth: a meta-regression analysis. World Dev..

[3] Jorgenson D.W., Vu K.M. (2016). The ICT revolution, world economic growth, and policy issues. Telecommun. Pol..

[4] Venturini F. (2015). The modern drivers of productivity. Res. Pol..

[5] Xu Q., Zhong M., Cao M. (2022). Does digital investment affect carbon efficiency? Spatial effect and mechanism discussion. Sci. Total Environ..

[6] Steinmueller W.E. (2001). ICTs and the possibilities for leapfrogging by developing countries. Int. Lab. Rev..

[7] Niebel T. (2018). ICT and economic growth - comparing developing, emerging and developed countries. World Dev..

[8] Afawubo K., Noglo Y.A. (2022). ICT and entrepreneurship: a comparative analysis of developing, emerging and developed countries. Technol. Forecast. Soc. Change.

[9] Ward M.R., Zheng S. (2016). Mobile telecommunications service and economic growth: evidence from China. Telecommun. Pol..

[10] Fakhimi M.A., Miremadi I. (2022). The impact of technological and social capabilities on innovation performance: a technological catch-up perspective. Technol. Soc..

[11] Black S.E., Lynch L.M. (2001). How to compete: the impact of workplace practices and information technology on productivity. Rev. Econ. Stat..

[12] Bresnahan T.F., Brynjolfsson E., Hitt L.M. (2002). Information technology, workplace organization, and the demand for skilled labor: firm-level evidence. Q. J. Econ..

[13] Luo K., Liu Y., Chen P.F., Zeng M. (2022). Assessing the impact of digital economy on green development efficiency in the Yangtze River Economic Belt. Energy Econ..

[14] Lucas Jr R.E. (1988). On the mechanics of economic development. J. Monetary Econ..

[15] Romer P.M. (1990). Endogenous technological change. J. Polit. Econ..

[16] Brynjolfsson E., Hitt L.M. (2000). Beyond computation: information technology, organizational transformation and business performance. J. Econ. Perspect..

[17] Hall B.H., Lotti F., Mairesse J. (2013). Evidence on the impact of R&D and ICT investments on innovation and productivity in Italian firms. Econ. Innovat. N. Technol..

[18] Haldar A., Sucharita S., Prasad Dash D., Sethi N., Chandra Padhan P. (2023). The effects of ICT, electricity consumption, innovation and renewable power generation on economic growth: an income level analysis for the emerging economies. J. Clean. Prod..

[19] Habibi F., Zabardast M.A. (2020). Digitalization, education and economic growth: a comparative analysis of Middle East and OECD countries. Technol. Soc..

[20] Myovella G., Karacuka M., Haucap J. (2020). Digitalization and economic growth: a comparative analysis of Sub-Saharan Africa and OECD economies. Telecommun. Pol..

[21] Cohen W.M., Levinthal D.A. (1989). Innovation and learning: the two faces of R&D. Econ. J..

[22] Leiponen A. (2005). Skills and innovation. Int. J. Ind. Organ..

[23] Abramovitz M. (1986). Catching up, forging ahead, and falling behind. J. Econ. Hist..

[24] Sterlacchini A. (2008). R&D, higher education and regional growth: uneven linkages among European regions. Res. Pol..

[25] Lee K. (2019).

[26] Banker R.D. (1993). Maximum-likelihood, consistency and data envelopment analysis: a statistical foundation. Manag. Sci..

[27] Simar L. (2007). How to improve the performances of DEA/FDH estimators in the presence of noise?. J. Prod. Anal..

[28] Zervopoulos P.D., Sklavos S., Kanas A., Cheng G. (2019). A multi-parametric method for bias correction of DEA efficiency estimators. J. Oper. Res. Soc..

[29] Fernández-Portillo A., Almodóvar-González M., Hernández-Mogollón R. (2020). Impact of ICT development on economic growth. A study of OECD European Union countries. Technol. Soc..

[30] Zhao N., Liu X., Pan C., Wang C. (2021). The performance of green innovation: from an efficiency perspective. Soc. Econ. Plann. Sci..

[31] Berkowitz P., Monfort P., Pieńkowski J. (2020). Unpacking the growth impacts of European Union Cohesion Policy: transmission channels from Cohesion Policy into economic growth. Reg. Stud..

[32] Lin B., Zhu J. (2019). The role of renewable energy technological innovation on climate change: empirical evidence from China. Sci. Total Environ..

[33] Zhou L., Shi T., Zhou Q. (2023). Is ICT development conducive to reducing the vulnerability of low-carbon energy? Evidence from OECD countries. Int. J. Environ. Res. Publ. Health.

[34] Edquist H., Henrekson M. (2017). Do R&D and ICT affect total factor productivity growth differently?. Telecommun. Pol..

[35] Thompson Jr H.G., Garbacz C. (2011). Economic impacts of mobile versus fixed broadband. Telecommun. Pol..

[36] Dewan S., Kraemer K.L. (2000). Information technology and productivity: evidence from country-level data. Manag. Sci..

[37] Colombo M.G., Croce A., Grilli L. (2013). ICT services and small businesses' productivity gains: an analysis of the adoption of broadband Internet technology. Inf. Econ. Pol..

[38] Bertschek I., Cerquera D., Klein G.J. (2013). More bits – more bucks? Measuring the impact of broadband internet on firm performance. Inf. Econ. Pol..

[39] Acemoglu D., Zilibotti F. (2001). Productivity differences. Q. J. Econ..

[40] Easterly W. (2019). In search of reforms for growth: new stylized facts on policy and growth outcomes. NBER Working Paper 26318.

[41] Nair M., Pradhan R.P., Arvin M.B. (2020). Endogenous dynamics between R&D, ICT and economic growth: empirical evidence from the OECD countries. Technol. Soc..

[42] Foote A. (2022). Comparing earnings outcome differences between all graduates and title IV graduates. Econ. Educ. Rev..

[43] van Ark B., Inklaar R., McGuckin R.H. (2003). ICT and productivity in Europe and the United States: where do the differences come from?. CESifo Econ. Stud..

[44] Becker G.S. (1962). Investment in human capital: a theoretical analysis. J. Polit. Econ..

[45] Goode R.B. (1959). Adding to the stock of physical and human capital. Am. Econ. Rev..

[46] Schultz T.W. (1961). Investment in human capital. Am. Econ. Rev..

[47] Liu C., Xia G. (2018). Research on the dynamic interrelationship among R&D investment, technological innovation, and economic growth in China. Sustainability.

[48] Gordon A.V., Ramic M., Rohrbeck R., Spaniol M.J. (2020). 50 Years of corporate and organizational foresight: looking back and going forward. Technol. Forecast. Soc. Change.

[49] Zhang D., Mohsin M., Rasheed A.K., Chang Y., Taghizadeh-Hesary F. (2021). Public spending and green economic growth in BRI region: mediating role of green finance. Energy Pol..

[50] Abu-Saad I. (2016). Access to higher education and its socio-economic impact among Bedouin Arabs in Southern Israel. Int. J. Educ. Res..

[51] Hall R.E., Jones C.I. (1999). Why do some countries produce so much more output per worker than others?. Q. J. Econ..

[52] Compagnucci L., Spigarelli F. (2020). The Third Mission of the university: a systematic literature review on potentials and constraints. Technol. Forecast. Soc. Change.

[53] Bilan Y., Mishchuk H., Roshchyk I., Kmecova I. (2020). An analysis of intellecutal potential and its impact on the social and economic development of European countries. Journal of Competitiveness.

[54] Cerquera D., Klein G. (2008). Endogenous firm heterogeneity, ICT and R&D incentives. ZEW – Centre for European Economic Research Discussion Paper No. 08-126.

[55] Polder M., van Leeuwen G., Mohnen P., Raymond W. (2009). Productivity effects of innovation modes. MPRA Paper No. 18893.

[56] Fernández-Portillo A., Almodóvar-González M., Sánchez-Escobedo M.C., Coca-Pérez J.L. (2022). The role of innovation in the relationship between digitalisation and economic and financial performance. A company-level research. European Research on Management and Business Economics.

[57] Greenan N., Mairesse J., Topiol-Bensaid A. (2001). Information technology and research and development impacts on productivity and skills: looking for correlations on French firm-level data. NBER Working Paper.

[58] Bugamelli M., Pagano P. (2004). Barriers to investment in ICT. Appl. Econ..

[59] Kneip A., Simar L., Wilson P.W. (2008). Asymptotics and consistent bootstraps for DEA estimators in nonparametric frontier models. Econom. Theor..

[60] Zervopoulos P.D., Triantis K., Sklavos S., Kanas A. (2023). An alternative Bayesian data envelopment analysis approach for correcting bias of efficiency estimators. J. Oper. Res. Soc..

[61] Cheng G., Zervopoulos P.D. (2014). Estimating the technical efficiency of health care systems: a cross-country comparison using the directional distance function. Eur. J. Oper. Res..

[62] Kounetas K., Zervopoulos P.D. (2019). A cross-country evaluation of environmental performance: is there a convergence-divergence pattern in technology gaps?. Eur. J. Oper. Res..

[63] Halkos G.E., Tzeremes N.G. (2010). Corruption and economic efficiency: panel data evidence. Global Econ. Rev..

[64] Kontolaimou A., Tsekouras K. (2010). Are cooperatives the weakest link in European banking? A non-parametric metafrontier approach. J. Bank. Finance.

[65] Poirier D.J. (1995).

[66] Ahn S.C., Schmidt P. (1995). Efficient estimation of models for dynamic panel data. J. Econom..

[67] Bun M.J.G., Sarafidis V., Baltagi B.H. (2015). The Oxford Handbook of Panel Data.

[68] Phillips P.C.B., Han C., Vinod H.D., Rao C.R. (2019). Handbook of Statistics: Conceptual Econometrics Using R.

[69] Swan T.W. (1956). Economic growth and capital accumulation. Econ. Rec..

[70] Czernich N., Falck O., Kretschmer T., Woessmann L. (2011). Broadband infrastructure and economic growth. Econ. J..

[71] Pradhan R.P., Arvin M.B., Norman N.R. (2015). The dynamics of information and communications technologies infrastructure, economic growth, and financial development: evidence from Asian countries. Technol. Soc..

[72] Choi C., Yi M.H. (2009). The effect of the Internet on economic growth: evidence from cross-country panel data. Econ. Lett..

[73] Ghosh S. (2016). Does mobile telephony spur growth? Evidence from Indian states. Telecommun. Pol..

[74] Arvanitis S., Loukis E.N. (2009). Information and communication technologies, human capital, workplace organization and labour productivity: a comparative study based on firm-level data for Greece and Switzerland. Inf. Econ. Pol..

[75] Easterly W. (2001). The lost decades: developing countries' stagnation in spite of policy reform 1980–1998. J. Econ. Growth.

[76] Lee S.H., Levendis J., Gutierrez L. (2012). Telecommunications and economic growth: an empirical analysis of sub-Saharan Africa. Appl. Econ..

[77] Lyu Y., Wang W., Wu Y., Zhang J. (2023). How does digital economy affect green total factor productivity? Evidence from China. Sci. Total Environ..

[78] Lee K. (2013).

