# Supplementary material for: Investigating the effects of ICT, education, and R&D on economic efficiency and technology heterogeneity: A cross-country analysis
Source: Heliyon. 2024 Mar 19;10(7):e28168. doi: 10.1016/j.heliyon.2024.e28168 (PMC10979069; doi:10.1016/j.heliyon.2024.e28168)
Supplement: Multimedia component 1 [file mmc1.docx]

**Electronic Supplement**

**Table ES1.** Descriptive statistics – Efficiencies

| Group | Statistics | Input | Input | Output |  | Bias-corrected Efficiencies | |  | Bias-corrected Metafrontier | |
| --- | --- | --- | --- | --- | --- | --- | --- | --- | --- | --- |
|  |  | Labor  (in millions) | Capital stock  (in millions of $, constant 2017) | GDP  (in millions of $, constant 2017) |  | Group-specific | Meta-efficiency |  | Technology Gap (TG) | Meta-technology ratio (MTR) |
| 2011 | | | | | | | | | | |
| All | Min | 0.182 | 47,253.44 | 13,687.66 |  | 0.261 | 0.248 |  | 0.013 | 0.822 |
|  | Max | 778.978 | 61,672,640.00 | 17,061,947.89 |  | 0.995 | 0.975 |  | 0.151 | 0.982 |
|  | Average | 30.241 | 6,119,194.59 | 1,361,157.40 |  | 0.721 | 0.653 |  | 0.068 | 0.907 |
|  | St. Dev. | 109.012 | 11,499,905.43 | 3,002,432.89 |  | 0.169 | 0.159 |  | 0.036 | 0.042 |
|  | N | 52 | 52 | 52 |  | 52 | 52 |  | 52 | 52 |
| Developed | Min | 0.182 | 47,253.44 | 13,687.66 |  | 0.363 | 0.336 |  | 0.015 | 0.822 |
|  | Max | 157.163 | 61,672,640.00 | 17,061,947.89 |  | 0.995 | 0.975 |  | 0.151 | 0.982 |
|  | Average | 15.740 | 6,948,123.93 | 1,451,807.32 |  | 0.728 | 0.665 |  | 0.064 | 0.913 |
|  | St. Dev. | 30.297 | 12,204,103.60 | 3,152,577.72 |  | 0.165 | 0.157 |  | 0.037 | 0.043 |
|  | N | 31 | 31 | 31 |  | 31 | 31 |  | 31 | 31 |
| Developing | Min | 0.942 | 83,560.61 | 23,833.24 |  | 0.261 | 0.248 |  | 0.013 | 0.841 |
|  | Max | 778.978 | 46,622,832.00 | 13,020,070.21 |  | 0.989 | 0.938 |  | 0.122 | 0.962 |
|  | Average | 51.648 | 4,895,536.99 | 1,227,340.86 |  | 0.710 | 0.636 |  | 0.074 | 0.898 |
|  | St. Dev. | 167.688 | 10,544,405.62 | 2,836,825.65 |  | 0.180 | 0.163 |  | 0.034 | 0.039 |
|  | N | 21 | 21 | 21 |  | 21 | 21 |  | 21 | 21 |
| 2012 | | | | | | | | | | |
| All | Min | 0.189 | 48,243.27 | 14,251.28 |  | 0.267 | 0.242 |  | 0.013 | 0.826 |
|  | Max | 782.865 | 62,435,632.00 | 17,445,764.23 |  | 0.996 | 0.960 |  | 0.145 | 0.980 |
|  | Average | 30.441 | 6,264,875.04 | 1,397,700.95 |  | 0.716 | 0.650 |  | 0.065 | 0.908 |
|  | St. Dev. | 109.545 | 11,876,007.54 | 3,124,725.08 |  | 0.168 | 0.158 |  | 0.033 | 0.039 |
|  | N | 52 | 52 | 52 |  | 52 | 52 |  | 52 | 52 |
| Developed | Min | 0.189 | 48,243.27 | 14,251.28 |  | 0.387 | 0.363 |  | 0.013 | 0.826 |
|  | Max | 157.842 | 62,435,632.00 | 17,445,764.23 |  | 0.985 | 0.960 |  | 0.145 | 0.980 |
|  | Average | 15.826 | 7,021,692.51 | 1,466,655.34 |  | 0.719 | 0.659 |  | 0.059 | 0.917 |
|  | St. Dev. | 30.402 | 12,330,612.46 | 3,218,678.47 |  | 0.162 | 0.157 |  | 0.031 | 0.039 |
|  | N | 31 | 31 | 31 |  | 31 | 31 |  | 31 | 31 |
| Developing | Min | 0.941 | 86,210.84 | 26,769.45 |  | 0.267 | 0.242 |  | 0.022 | 0.839 |
|  | Max | 782.865 | 50,743,640.00 | 14,043,934.22 |  | 0.996 | 0.939 |  | 0.131 | 0.964 |
|  | Average | 52.014 | 5,147,668.31 | 1,295,911.15 |  | 0.711 | 0.637 |  | 0.075 | 0.896 |
|  | St. Dev. | 168.506 | 11,374,818.85 | 3,056,121.21 |  | 0.180 | 0.164 |  | 0.033 | 0.036 |
|  | N | 21 | 21 | 21 |  | 21 | 21 |  | 21 | 21 |
| 2013 | | | | | | | | | | |
| All | Min | 0.197 | 49,214.75 | 15,031.31 |  | 0.264 | 0.249 |  | 0.000 | 0.822 |
|  | Max | 786.673 | 63,232,936.00 | 17,767,129.35 |  | 0.984 | 0.976 |  | 0.138 | 1.000 |
|  | Average | 30.620 | 6,429,900.19 | 1,437,112.59 |  | 0.710 | 0.652 |  | 0.059 | 0.918 |
|  | St. Dev. | 110.064 | 12,349,130.59 | 3,248,357.59 |  | 0.167 | 0.159 |  | 0.034 | 0.042 |
|  | N | 52 | 52 | 52 |  | 52 | 52 |  | 52 | 52 |
| Developed | Min | 0.197 | 49,214.75 | 15,031.31 |  | 0.396 | 0.372 |  | 0.000 | 0.870 |
|  | Max | 158.253 | 63,232,936.00 | 17,767,129.35 |  | 0.984 | 0.976 |  | 0.093 | 1.000 |
|  | Average | 15.901 | 7,094,408.76 | 1,485,541.14 |  | 0.709 | 0.662 |  | 0.047 | 0.933 |
|  | St. Dev. | 30.501 | 12,463,165.79 | 3,275,967.84 |  | 0.164 | 0.161 |  | 0.029 | 0.037 |
|  | N | 31 | 31 | 31 |  | 31 | 31 |  | 31 | 31 |
| Developing | Min | 0.955 | 88,388.22 | 29,121.20 |  | 0.264 | 0.249 |  | 0.016 | 0.822 |
|  | Max | 786.673 | 55,869,636.00 | 15,134,607.23 |  | 0.974 | 0.923 |  | 0.138 | 0.974 |
|  | Average | 52.349 | 5,448,958.98 | 1,365,622.83 |  | 0.712 | 0.637 |  | 0.076 | 0.895 |
|  | St. Dev. | 169.304 | 12,417,208.64 | 3,286,367.20 |  | 0.176 | 0.159 |  | 0.035 | 0.040 |
|  | N | 21 | 21 | 21 |  | 21 | 21 |  | 21 | 21 |
| 2014 | | | | | | | | | | |
| All | Min | 0.206 | 50,358.89 | 16,178.73 |  | 0.244 | 0.228 |  | 0.000 | 0.842 |
|  | Max | 791.324 | 64,124,208.00 | 18,215,922.32 |  | 0.993 | 0.982 |  | 0.152 | 1.000 |
|  | Average | 30.811 | 6,594,588.07 | 1,481,650.62 |  | 0.708 | 0.658 |  | 0.050 | 0.930 |
|  | St. Dev. | 110.700 | 12,838,285.51 | 3,389,244.41 |  | 0.169 | 0.160 |  | 0.032 | 0.037 |
|  | N | 52 | 52 | 52 |  | 52 | 52 |  | 52 | 52 |
| Developed | Min | 0.206 | 50,358.89 | 16,178.73 |  | 0.396 | 0.383 |  | 0.000 | 0.889 |
|  | Max | 158.910 | 64,124,208.00 | 18,215,922.32 |  | 0.987 | 0.982 |  | 0.083 | 1.000 |
|  | Average | 15.982 | 7,173,508.05 | 1,515,422.24 |  | 0.709 | 0.670 |  | 0.039 | 0.944 |
|  | St. Dev. | 30.628 | 12,612,577.35 | 3,353,553.60 |  | 0.164 | 0.161 |  | 0.023 | 0.030 |
|  | N | 31 | 31 | 31 |  | 31 | 31 |  | 31 | 31 |
| Developing | Min | 0.960 | 90,359.99 | 30,178.05 |  | 0.244 | 0.228 |  | 0.017 | 0.842 |
|  | Max | 791.324 | 60,808,008.00 | 16,258,467.39 |  | 0.993 | 0.936 |  | 0.152 | 0.966 |
|  | Average | 52.702 | 5,739,991.92 | 1,431,797.26 |  | 0.706 | 0.641 |  | 0.066 | 0.909 |
|  | St. Dev. | 170.288 | 13,431,047.01 | 3,523,908.77 |  | 0.180 | 0.162 |  | 0.038 | 0.038 |
|  | N | 21 | 21 | 21 |  | 21 | 21 |  | 21 | 21 |
| 2015 | | | | | | | | | | |
| All | Min | 0.213 | 53,435.04 | 17,733.20 |  | 0.221 | 0.206 |  | 0.000 | 0.869 |
|  | Max | 795.251 | 65,057,808.00 | 18,776,155.68 |  | 0.984 | 0.969 |  | 0.111 | 1.000 |
|  | Average | 30.997 | 6,744,846.28 | 1,530,120.81 |  | 0.707 | 0.662 |  | 0.045 | 0.937 |
|  | St. Dev. | 111.250 | 13,265,989.01 | 3,544,057.72 |  | 0.171 | 0.163 |  | 0.029 | 0.036 |
|  | N | 52 | 52 | 52 |  | 52 | 52 |  | 52 | 52 |
| Developed | Min | 0.213 | 53,435.04 | 17,733.20 |  | 0.397 | 0.388 |  | 0.000 | 0.869 |
|  | Max | 159.913 | 65,057,808.00 | 18,776,155.68 |  | 0.982 | 0.969 |  | 0.094 | 1.000 |
|  | Average | 16.068 | 7,259,401.53 | 1,553,016.26 |  | 0.715 | 0.678 |  | 0.037 | 0.948 |
|  | St. Dev. | 30.808 | 12,769,257.41 | 3,450,571.46 |  | 0.167 | 0.160 |  | 0.026 | 0.031 |
|  | N | 31 | 31 | 31 |  | 31 | 31 |  | 31 | 31 |
| Developing | Min | 0.953 | 92,338.03 | 31,341.67 |  | 0.221 | 0.206 |  | 0.010 | 0.873 |
|  | Max | 795.251 | 64,797,304.00 | 17,403,279.55 |  | 0.984 | 0.938 |  | 0.111 | 0.989 |
|  | Average | 53.035 | 5,985,264.72 | 1,496,322.76 |  | 0.695 | 0.639 |  | 0.056 | 0.920 |
|  | St. Dev. | 171.118 | 14,253,714.63 | 3,763,934.51 |  | 0.180 | 0.168 |  | 0.031 | 0.036 |
|  | N | 21 | 21 | 21 |  | 21 | 21 |  | 21 | 21 |
| 2016 | | | | | | | | | | |
| All | Min | 0.222 | 56,173.43 | 18,458.65 |  | 0.219 | 0.218 |  | 0.000 | 0.897 |
|  | Max | 797.668 | 68,728,456.00 | 19,097,495.83 |  | 0.994 | 0.976 |  | 0.101 | 1.000 |
|  | Average | 31.189 | 6,895,045.94 | 1,574,715.33 |  | 0.706 | 0.669 |  | 0.038 | 0.948 |
|  | St. Dev. | 111.622 | 13,698,051.54 | 3,683,074.15 |  | 0.170 | 0.162 |  | 0.024 | 0.028 |
|  | N | 52 | 52 | 52 |  | 52 | 52 |  | 52 | 52 |
| Developed | Min | 0.222 | 56,173.43 | 18,458.65 |  | 0.416 | 0.401 |  | 0.004 | 0.897 |
|  | Max | 161.836 | 65,974,060.00 | 19,097,495.83 |  | 0.994 | 0.976 |  | 0.101 | 0.995 |
|  | Average | 16.220 | 7,348,584.32 | 1,580,567.97 |  | 0.719 | 0.685 |  | 0.034 | 0.953 |
|  | St. Dev. | 31.163 | 12,924,579.39 | 3,508,134.19 |  | 0.166 | 0.157 |  | 0.022 | 0.025 |
|  | N | 31 | 31 | 31 |  | 31 | 31 |  | 31 | 31 |
| Developing | Min | 0.947 | 93,674.64 | 32,234.35 |  | 0.219 | 0.218 |  | 0.000 | 0.898 |
|  | Max | 797.668 | 68,728,456.00 | 18,595,188.78 |  | 0.978 | 0.971 |  | 0.094 | 1.000 |
|  | Average | 53.286 | 6,225,536.89 | 1,566,075.72 |  | 0.688 | 0.645 |  | 0.042 | 0.940 |
|  | St. Dev. | 171.625 | 15,070,393.41 | 4,016,242.04 |  | 0.178 | 0.169 |  | 0.026 | 0.031 |
|  | N | 21 | 21 | 21 |  | 21 | 21 |  | 21 | 21 |
| 2017 | | | | | | | | | | |
| All | Min | 0.232 | 58,251.33 | 19,957.39 |  | 0.230 | 0.222 |  | 0.000 | 0.911 |
|  | Max | 799.175 | 72,763,856.00 | 19,887,033.88 |  | 0.988 | 0.979 |  | 0.088 | 1.000 |
|  | Average | 31.334 | 7,054,193.53 | 1,631,727.69 |  | 0.706 | 0.682 |  | 0.025 | 0.965 |
|  | St. Dev. | 111.859 | 14,154,938.35 | 3,846,801.87 |  | 0.170 | 0.165 |  | 0.018 | 0.022 |
|  | N | 52 | 52 | 52 |  | 52 | 52 |  | 52 | 52 |
| Developed | Min | 0.232 | 58,251.33 | 19,957.39 |  | 0.412 | 0.409 |  | 0.000 | 0.938 |
|  | Max | 163.395 | 66,942,704.00 | 19,542,979.18 |  | 0.988 | 0.979 |  | 0.044 | 1.000 |
|  | Average | 16.339 | 7,446,215.62 | 1,619,678.34 |  | 0.719 | 0.700 |  | 0.019 | 0.973 |
|  | St. Dev. | 31.444 | 13,091,488.70 | 3,588,257.17 |  | 0.161 | 0.159 |  | 0.013 | 0.017 |
|  | N | 31 | 31 | 31 |  | 31 | 31 |  | 31 | 31 |
| Developing | Min | 0.953 | 95,323.90 | 32,583.05 |  | 0.230 | 0.222 |  | 0.000 | 0.911 |
|  | Max | 799.175 | 72,763,856.00 | 19,887,033.88 |  | 0.987 | 0.977 |  | 0.088 | 1.000 |
|  | Average | 53.468 | 6,475,494.25 | 1,649,514.83 |  | 0.688 | 0.654 |  | 0.034 | 0.952 |
|  | St. Dev. | 171.933 | 15,913,935.88 | 4,291,930.14 |  | 0.185 | 0.174 |  | 0.022 | 0.024 |
|  | N | 21 | 21 | 21 |  | 21 | 21 |  | 21 | 21 |
| 2018 | | | | | | | | | | |
| All | Min | 0.250 | 60,106.05 | 20,991.02 |  | 0.233 | 0.232 |  | 0.000 | 0.956 |
|  | Max | 799.480 | 76,689,208.00 | 21,229,363.69 |  | 0.992 | 0.990 |  | 0.043 | 1.000 |
|  | Average | 31.451 | 7,213,576.04 | 1,688,553.09 |  | 0.700 | 0.691 |  | 0.012 | 0.984 |
|  | St. Dev. | 111.936 | 14,617,463.55 | 4,027,726.68 |  | 0.169 | 0.166 |  | 0.011 | 0.014 |
|  | N | 52 | 52 | 52 |  | 52 | 52 |  | 52 | 52 |
| Developed | Min | 0.250 | 60,106.05 | 20,991.02 |  | 0.425 | 0.428 |  | 0.000 | 0.965 |
|  | Max | 164.635 | 68,007,352.00 | 20,128,577.59 |  | 0.992 | 0.990 |  | 0.033 | 1.000 |
|  | Average | 16.462 | 7,546,969.49 | 1,656,478.04 |  | 0.714 | 0.709 |  | 0.008 | 0.988 |
|  | St. Dev. | 31.701 | 13,274,443.48 | 3,687,949.21 |  | 0.159 | 0.157 |  | 0.010 | 0.013 |
|  | N | 31 | 31 | 31 |  | 31 | 31 |  | 31 | 31 |
| Developing | Min | 0.957 | 97,052.55 | 33,521.64 |  | 0.233 | 0.232 |  | 0.000 | 0.956 |
|  | Max | 799.480 | 76,689,208.00 | 21,229,363.69 |  | 0.987 | 0.963 |  | 0.043 | 1.000 |
|  | Average | 53.578 | 6,721,423.80 | 1,735,901.97 |  | 0.680 | 0.664 |  | 0.016 | 0.977 |
|  | St. Dev. | 171.990 | 16,736,673.22 | 4,578,447.54 |  | 0.185 | 0.179 |  | 0.011 | 0.014 |
|  | N | 21 | 21 | 21 |  | 21 | 21 |  | 21 | 21 |
| 2019 | | | | | | | | | | |
| All | Min | 0.266 | 61,996.11 | 22,153.84 |  | 0.235 | 0.242 |  | 0.000 | 0.941 |
|  | Max | 800.021 | 81,726,344.00 | 22,492,450.17 |  | 0.993 | 0.995 |  | 0.027 | 1.000 |
|  | Average | 31.565 | 7,393,674.07 | 1,735,972.35 |  | 0.698 | 0.698 |  | 0.005 | 0.993 |
|  | St. Dev. | 112.047 | 15,190,194.06 | 4,188,742.57 |  | 0.169 | 0.168 |  | 0.007 | 0.011 |
|  | N | 52 | 52 | 52 |  | 52 | 52 |  | 52 | 52 |
| Developed | Min | 0.266 | 61,996.11 | 22,153.84 |  | 0.424 | 0.428 |  | 0.000 | 0.941 |
|  | Max | 166.356 | 69,059,072.00 | 20,563,591.68 |  | 0.993 | 0.992 |  | 0.027 | 1.000 |
|  | Average | 16.594 | 7,650,704.86 | 1,684,859.91 |  | 0.717 | 0.716 |  | 0.005 | 0.992 |
|  | St. Dev. | 32.018 | 13,454,967.54 | 3,762,541.49 |  | 0.155 | 0.156 |  | 0.008 | 0.013 |
|  | N | 31 | 31 | 31 |  | 31 | 31 |  | 31 | 31 |
| Developing | Min | 0.963 | 98,812.79 | 34,832.47 |  | 0.235 | 0.242 |  | 0.000 | 0.977 |
|  | Max | 800.021 | 81,726,344.00 | 22,492,450.17 |  | 0.993 | 0.995 |  | 0.023 | 1.000 |
|  | Average | 53.665 | 7,014,247.66 | 1,811,424.05 |  | 0.670 | 0.672 |  | 0.004 | 0.995 |
|  | St. Dev. | 172.094 | 17,792,815.93 | 4,847,275.97 |  | 0.188 | 0.185 |  | 0.006 | 0.008 |
|  | N | 21 | 21 | 21 |  | 21 | 21 |  | 21 | 21 |

**Table ES2.** Descriptive statistics – Regression variables

| Group | Statistics | Education proxy | ICT proxies | | | Innovation proxy | Control variables | | |
| --- | --- | --- | --- | --- | --- | --- | --- | --- | --- |
|  |  | Education expenditure  (% of GNI) | Fixed broadband subscriptions  (per 100 people) | Individuals using the Internet  (% of population) | Mobile cellular subscriptions  (per 100 people) | Research and development (R&D) expenditure  (% of GDP) | Trade  (% of GDP) | Consumer price index  (annual change %) |  |
| 2011 | | | | | | | | |  |
| All | Min | 1.790 | 1.744 | 12.500 | 71.649 | 0.036 | 30.195 | -0.003 |  |
|  | Max | 8.070 | 38.821 | 93.490 | 218.265 | 4.003 | 421.855 | 0.532 |  |
|  | Average | 4.627 | 21.336 | 61.804 | 121.540 | 1.401 | 113.270 | 0.051 |  |
|  | St. Dev. | 1.238 | 10.578 | 20.659 | 24.813 | 1.039 | 82.400 | 0.072 |  |
|  | N | 52 | 52 | 52 | 52 | 52 | 52 | 52 |  |
| Developed | Min | 2.808 | 17.621 | 51.650 | 77.709 | 0.454 | 30.195 | -0.003 |  |
|  | Max | 8.070 | 38.821 | 93.490 | 218.265 | 4.003 | 421.855 | 0.053 |  |
|  | Average | 4.951 | 28.460 | 75.128 | 124.850 | 1.942 | 132.571 | 0.032 |  |
|  | St. Dev. | 1.270 | 5.614 | 11.630 | 26.961 | 0.991 | 98.702 | 0.011 |  |
|  | N | 31 | 31 | 31 | 31 | 31 | 31 | 31 |  |
| Developing | Min | 1.790 | 1.744 | 12.500 | 71.649 | 0.036 | 39.470 | 0.023 |  |
|  | Max | 6.013 | 23.375 | 68.020 | 153.061 | 1.780 | 166.366 | 0.532 |  |
|  | Average | 4.148 | 10.820 | 42.134 | 116.655 | 0.603 | 84.777 | 0.081 |  |
|  | St. Dev. | 1.041 | 6.579 | 14.282 | 20.919 | 0.398 | 35.650 | 0.106 |  |
|  | N | 21 | 21 | 21 | 21 | 21 | 21 | 21 |  |
| 2012 | | | | | | | | |  |
| All | Min | 1.790 | 2.096 | 16.400 | 79.377 | 0.034 | 30.471 | 0.000 |  |
|  | Max | 7.240 | 39.626 | 94.650 | 232.551 | 4.142 | 430.569 | 0.592 |  |
|  | Average | 4.706 | 22.265 | 64.702 | 125.509 | 1.424 | 114.236 | 0.044 |  |
|  | St. Dev. | 1.243 | 10.508 | 19.559 | 26.546 | 1.050 | 82.956 | 0.081 |  |
|  | N | 52 | 52 | 52 | 52 | 52 | 52 | 52 |  |
| Developed | Min | 2.838 | 19.308 | 55.070 | 79.377 | 0.439 | 30.471 | 0.000 |  |
|  | Max | 7.240 | 39.626 | 94.650 | 232.551 | 4.142 | 430.569 | 0.046 |  |
|  | Average | 4.979 | 29.250 | 77.028 | 127.951 | 1.960 | 134.598 | 0.024 |  |
|  | St. Dev. | 1.259 | 5.484 | 10.967 | 28.927 | 1.010 | 99.136 | 0.010 |  |
|  | N | 31 | 31 | 31 | 31 | 31 | 31 | 31 |  |
| Developing | Min | 1.790 | 2.096 | 16.400 | 80.346 | 0.034 | 38.835 | 0.006 |  |
|  | Max | 6.312 | 26.886 | 70.580 | 180.493 | 1.912 | 165.593 | 0.592 |  |
|  | Average | 4.303 | 11.954 | 46.507 | 121.904 | 0.634 | 84.178 | 0.073 |  |
|  | St. Dev. | 1.131 | 7.016 | 14.561 | 22.779 | 0.426 | 34.860 | 0.122 |  |
|  | N | 21 | 21 | 21 | 21 | 21 | 21 | 21 |  |
| 2013 | | | | | | | | |  |
| All | Min | 1.790 | 3.009 | 17.700 | 80.348 | 0.064 | 30.002 | -0.009 |  |
|  | Max | 7.731 | 40.324 | 95.053 | 239.437 | 4.075 | 442.620 | 0.183 |  |
|  | Average | 4.676 | 23.331 | 67.289 | 125.303 | 1.436 | 112.778 | 0.027 |  |
|  | St. Dev. | 1.253 | 10.597 | 18.969 | 25.953 | 1.047 | 83.311 | 0.033 |  |
|  | N | 52 | 52 | 52 | 52 | 52 | 52 | 52 |  |
| Developed | Min | 2.760 | 20.456 | 58.459 | 80.348 | 0.486 | 30.002 | -0.009 |  |
|  | Max | 7.731 | 40.324 | 95.053 | 239.437 | 4.075 | 442.620 | 0.043 |  |
|  | Average | 4.968 | 30.324 | 79.065 | 126.721 | 1.966 | 134.103 | 0.013 |  |
|  | St. Dev. | 1.289 | 5.359 | 10.718 | 27.861 | 1.008 | 99.782 | 0.010 |  |
|  | N | 31 | 31 | 31 | 31 | 31 | 31 | 31 |  |
| Developing | Min | 1.790 | 3.009 | 17.700 | 88.306 | 0.064 | 37.987 | -0.002 |  |
|  | Max | 6.326 | 29.563 | 72.644 | 178.343 | 1.998 | 164.288 | 0.183 |  |
|  | Average | 4.244 | 13.007 | 49.905 | 123.210 | 0.654 | 81.299 | 0.049 |  |
|  | St. Dev. | 1.086 | 7.453 | 14.593 | 23.355 | 0.439 | 31.916 | 0.043 |  |
|  | N | 21 | 21 | 21 | 21 | 21 | 21 | 21 |  |
| 2014 | | | | | | | | |  |
| All | Min | 1.790 | 3.128 | 19.942 | 80.722 | 0.094 | 29.998 | -0.014 |  |
|  | Max | 7.042 | 41.343 | 96.300 | 237.705 | 4.155 | 425.976 | 0.181 |  |
|  | Average | 4.597 | 24.166 | 69.735 | 125.394 | 1.445 | 112.958 | 0.024 |  |
|  | St. Dev. | 1.206 | 10.720 | 18.050 | 24.181 | 1.048 | 81.688 | 0.039 |  |
|  | N | 52 | 52 | 52 | 52 | 52 | 52 | 52 |  |
| Developed | Min | 2.760 | 21.943 | 55.638 | 80.722 | 0.514 | 29.998 | -0.014 |  |
|  | Max | 7.042 | 41.343 | 96.300 | 237.705 | 4.155 | 425.976 | 0.044 |  |
|  | Average | 4.849 | 31.367 | 80.729 | 126.552 | 1.973 | 133.710 | 0.007 |  |
|  | St. Dev. | 1.226 | 5.264 | 10.065 | 25.883 | 1.013 | 97.541 | 0.011 |  |
|  | N | 31 | 31 | 31 | 31 | 31 | 31 | 31 |  |
| Developing | Min | 1.790 | 3.128 | 19.942 | 87.199 | 0.094 | 36.920 | -0.014 |  |
|  | Max | 5.970 | 28.461 | 75.653 | 165.268 | 2.022 | 168.341 | 0.181 |  |
|  | Average | 4.224 | 13.536 | 53.506 | 123.684 | 0.667 | 82.324 | 0.050 |  |
|  | St. Dev. | 1.098 | 7.243 | 14.661 | 21.932 | 0.441 | 33.041 | 0.051 |  |
|  | N | 21 | 21 | 21 | 21 | 21 | 21 | 21 |  |
| 2015 | | | | | | | | |  |
| All | Min | 1.790 | 2.545 | 22.500 | 82.619 | 0.143 | 27.812 | -0.021 |  |
|  | Max | 7.010 | 42.276 | 96.810 | 232.737 | 4.261 | 389.406 | 0.487 |  |
|  | Average | 4.540 | 25.257 | 71.893 | 125.040 | 1.455 | 111.390 | 0.027 |  |
|  | St. Dev. | 1.119 | 10.880 | 17.138 | 23.693 | 1.039 | 79.631 | 0.074 |  |
|  | N | 52 | 52 | 52 | 52 | 52 | 52 | 52 |  |
| Developed | Min | 2.760 | 23.435 | 58.142 | 82.619 | 0.477 | 27.812 | -0.021 |  |
|  | Max | 7.010 | 42.276 | 96.810 | 232.737 | 4.261 | 389.406 | 0.030 |  |
|  | Average | 4.812 | 32.443 | 82.088 | 127.189 | 1.974 | 132.201 | 0.002 |  |
|  | St. Dev. | 1.123 | 5.398 | 9.502 | 24.728 | 1.005 | 94.393 | 0.010 |  |
|  | N | 31 | 31 | 31 | 31 | 31 | 31 | 31 |  |
| Developing | Min | 1.790 | 2.545 | 22.500 | 88.372 | 0.143 | 34.846 | -0.009 |  |
|  | Max | 5.900 | 30.759 | 76.630 | 158.883 | 2.057 | 167.272 | 0.487 |  |
|  | Average | 4.137 | 14.650 | 56.844 | 121.869 | 0.689 | 80.669 | 0.063 |  |
|  | St. Dev. | 1.007 | 7.744 | 14.651 | 22.282 | 0.454 | 33.799 | 0.107 |  |
|  | N | 21 | 21 | 21 | 21 | 21 | 21 | 21 |  |
| 2016 | | | | | | | | |  |
| All | Min | 1.790 | 2.047 | 22.266 | 84.523 | 0.142 | 26.587 | -0.014 |  |
|  | Max | 7.205 | 43.097 | 98.137 | 242.768 | 4.511 | 371.727 | 0.145 |  |
|  | Average | 4.499 | 26.298 | 74.339 | 124.839 | 1.423 | 110.440 | 0.021 |  |
|  | St. Dev. | 1.139 | 11.137 | 16.619 | 23.349 | 1.067 | 77.699 | 0.040 |  |
|  | N | 52 | 52 | 52 | 52 | 52 | 52 | 52 |  |
| Developed | Min | 2.740 | 24.560 | 61.324 | 84.523 | 0.435 | 26.587 | -0.014 |  |
|  | Max | 7.205 | 43.097 | 98.137 | 242.768 | 4.511 | 371.727 | 0.036 |  |
|  | Average | 4.775 | 33.610 | 83.752 | 127.437 | 1.939 | 130.014 | 0.005 |  |
|  | St. Dev. | 1.175 | 5.398 | 8.789 | 25.418 | 1.056 | 92.020 | 0.010 |  |
|  | N | 31 | 31 | 31 | 31 | 31 | 31 | 31 |  |
| Developing | Min | 1.790 | 2.047 | 22.266 | 90.592 | 0.142 | 30.247 | -0.014 |  |
|  | Max | 5.900 | 32.476 | 83.559 | 157.718 | 2.100 | 164.370 | 0.145 |  |
|  | Average | 4.092 | 15.506 | 60.444 | 121.004 | 0.663 | 81.546 | 0.045 |  |
|  | St. Dev. | 0.972 | 8.212 | 15.758 | 19.879 | 0.457 | 34.964 | 0.053 |  |
|  | N | 21 | 21 | 21 | 21 | 21 | 21 | 21 |  |
| 2017 | | | | | | | | |  |
| All | Min | 1.790 | 1.970 | 23.714 | 86.281 | 0.127 | 27.246 | 0.002 |  |
|  | Max | 7.165 | 43.922 | 97.363 | 251.765 | 4.657 | 376.796 | 0.295 |  |
|  | Average | 4.409 | 27.222 | 76.156 | 125.605 | 1.449 | 114.125 | 0.032 |  |
|  | St. Dev. | 1.111 | 11.177 | 15.643 | 24.216 | 1.090 | 77.805 | 0.045 |  |
|  | N | 52 | 52 | 52 | 52 | 52 | 52 | 52 |  |
| Developed | Min | 2.719 | 25.801 | 63.077 | 86.281 | 0.511 | 27.246 | 0.002 |  |
|  | Max | 7.165 | 43.922 | 97.363 | 251.765 | 4.657 | 376.796 | 0.037 |  |
|  | Average | 4.639 | 34.355 | 85.160 | 128.247 | 1.975 | 133.247 | 0.016 |  |
|  | St. Dev. | 1.144 | 5.706 | 7.998 | 27.012 | 1.077 | 92.100 | 0.008 |  |
|  | N | 31 | 31 | 31 | 31 | 31 | 31 | 31 |  |
| Developing | Min | 1.790 | 1.970 | 23.714 | 91.627 | 0.127 | 35.283 | 0.010 |  |
|  | Max | 5.900 | 33.473 | 82.327 | 156.187 | 2.116 | 165.201 | 0.295 |  |
|  | Average | 4.068 | 16.693 | 62.865 | 121.706 | 0.673 | 85.896 | 0.055 |  |
|  | St. Dev. | 0.990 | 8.630 | 14.727 | 19.342 | 0.477 | 36.189 | 0.065 |  |
|  | N | 21 | 21 | 21 | 21 | 21 | 21 | 21 |  |
| 2018 | | | | | | | | |  |
| All | Min | 1.790 | 1.915 | 43.800 | 89.579 | 0.102 | 27.589 | 0.004 |  |
|  | Max | 7.165 | 44.776 | 97.319 | 269.934 | 4.797 | 376.893 | 0.163 |  |
|  | Average | 4.413 | 28.032 | 79.021 | 125.721 | 1.484 | 116.294 | 0.030 |  |
|  | St. Dev. | 1.112 | 11.226 | 12.842 | 26.109 | 1.116 | 77.093 | 0.031 |  |
|  | N | 52 | 52 | 52 | 52 | 52 | 52 | 52 |  |
| Developed | Min | 2.719 | 25.942 | 72.238 | 89.579 | 0.593 | 27.589 | 0.004 |  |
|  | Max | 7.165 | 44.776 | 97.319 | 269.934 | 4.797 | 376.893 | 0.034 |  |
|  | Average | 4.639 | 35.144 | 86.570 | 127.726 | 2.017 | 134.678 | 0.017 |  |
|  | St. Dev. | 1.144 | 5.818 | 6.636 | 29.913 | 1.104 | 91.427 | 0.007 |  |
|  | N | 31 | 31 | 31 | 31 | 31 | 31 | 31 |  |
| Developing | Min | 1.790 | 1.915 | 43.800 | 94.534 | 0.102 | 36.535 | 0.011 |  |
|  | Max | 5.900 | 33.869 | 84.900 | 159.931 | 2.141 | 163.239 | 0.163 |  |
|  | Average | 4.080 | 17.533 | 67.876 | 122.761 | 0.696 | 89.157 | 0.049 |  |
|  | St. Dev. | 0.996 | 8.745 | 11.650 | 19.510 | 0.506 | 36.330 | 0.042 |  |
|  | N | 21 | 21 | 21 | 21 | 21 | 21 | 21 |  |
| 2019 | | | | | | | | |  |
| All | Min | 1.790 | 2.135 | 50.491 | 91.863 | 0.093 | 26.369 | 0.001 |  |
|  | Max | 7.165 | 45.987 | 98.046 | 288.533 | 5.140 | 380.104 | 0.152 |  |
|  | Average | 4.419 | 28.902 | 81.538 | 127.785 | 1.529 | 115.955 | 0.026 |  |
|  | St. Dev. | 1.118 | 11.294 | 11.581 | 28.838 | 1.157 | 77.899 | 0.027 |  |
|  | N | 52 | 52 | 52 | 52 | 52 | 52 | 52 |  |
| Developed | Min | 2.719 | 25.912 | 75.346 | 91.863 | 0.589 | 26.369 | 0.003 |  |
|  | Max | 7.165 | 45.987 | 98.046 | 288.533 | 5.140 | 380.104 | 0.029 |  |
|  | Average | 4.639 | 35.958 | 88.269 | 130.058 | 2.079 | 134.902 | 0.015 |  |
|  | St. Dev. | 1.144 | 6.014 | 6.333 | 32.975 | 1.144 | 92.349 | 0.008 |  |
|  | N | 31 | 31 | 31 | 31 | 31 | 31 | 31 |  |
| Developing | Min | 1.790 | 2.135 | 50.491 | 92.203 | 0.093 | 35.890 | 0.001 |  |
|  | Max | 5.900 | 34.011 | 86.100 | 165.600 | 2.245 | 161.236 | 0.152 |  |
|  | Average | 4.094 | 18.487 | 71.603 | 124.430 | 0.716 | 87.985 | 0.044 |  |
|  | St. Dev. | 1.020 | 8.942 | 10.392 | 21.677 | 0.536 | 36.104 | 0.035 |  |
|  | N | 21 | 21 | 21 | 21 | 21 | 21 | 21 |  |
